# Proteome Changes Reveal the Protective Roles of Exogenous Citric Acid in Alleviating Cu Toxicity in *Brassica napus* L.

**DOI:** 10.3390/ijms22115879

**Published:** 2021-05-30

**Authors:** Young-Hwan Ju, Swapan Kumar Roy, Aritra Roy Choudhury, Soo-Jeong Kwon, Ju-Young Choi, Md Atikur Rahman, Tomoyuki Katsube-Tanaka, Tatsuhiko Shiraiwa, Moon-Soon Lee, Kun Cho, Sun-Hee Woo

**Affiliations:** 1 Department of Crop Science, Chungbuk National University, Cheong-ju 28644, Korea; joh7598@naver.com (Y.-H.J.); roy@chungbuk.ac.kr (S.K.R.); kwonsj1220@naver.com (S.-J.K.); jychoi8519@gmail.com (J.-Y.C.); 2 College of Agricultural Sciences, IUBAT—International University of Business Agriculture and Technology, 4 Embankment Drive Road, Sector 10 Uttara Model Town, Dhaka 1230, Bangladesh; 3 Department of Environmental and Biological Chemistry, Chungbuk National University, Cheong-ju 28644, Korea; aritraroy@chungbuk.ac.kr; 4 Grassland and Forage Division, Rural Development Administration, National Institute of Animal Science, Cheonan 31000, Korea; atikbt@korea.kr; 5 Graduate School of Agriculture, Kyoto University, Kitashirakawa Oiwake-cho, Sakyo-ku, Kyoto 606-8502, Japan; tanakato@kais.kyoto-u.ac.jp (T.K.-T.); shiraiwa@kais.kyoto-u.ac.jp (T.S.); 6 Department of Industrial Plant Science & Technology, Chungbuk National University, Cheong-ju 28644, Korea; mslee416@chungbuk.ac.kr; 7 Bio-Chemical Analysis Team, Center for Research Equipment, Korea Basic Science Institute, Ochang, Cheong-ju 28119, Korea

**Keywords:** Cu toxicity, citric acid, *Brassica napus*, proteomics, phytoextraction, plant growth

## Abstract

Citric acid (CA), as an organic chelator, plays a vital role in alleviating copper (Cu) stress-mediated oxidative damage, wherein a number of molecular mechanisms alter in plants. However, it remains largely unknown how CA regulates differentially abundant proteins (DAPs) in response to Cu stress in *Brassica napus* L. In the present study, we aimed to investigate the proteome changes in the leaves of *B.* L. seedlings in response to CA-mediated alleviation of Cu stress. Exposure of 21-day-old seedlings to Cu (25 and 50 μM) and CA (1.0 mM) for 7 days exhibited a dramatic inhibition of overall growth and considerable increase in the enzymatic activities (POD, SOD, CAT). Using a label-free proteome approach, a total of 6345 proteins were identified in differentially treated leaves, from which 426 proteins were differentially expressed among the treatment groups. Gene ontology (GO) and KEGG pathways analysis revealed that most of the differential abundance proteins were found to be involved in energy and carbohydrate metabolism, photosynthesis, protein metabolism, stress and defense, metal detoxification, and cell wall reorganization. Our results suggest that the downregulation of chlorophyll biosynthetic proteins involved in photosynthesis were consistent with reduced chlorophyll content. The increased abundance of proteins involved in stress and defense indicates that these DAPs might provide significant insights into the adaptation of Brassica seedlings to Cu stress. The abundances of key proteins were further verified by monitoring the mRNA expression level of the respective transcripts. Taken together, these findings provide a potential molecular mechanism towards Cu stress tolerance and open a new route in accelerating the phytoextraction of Cu through exogenous application of CA in *B. napus*.

## 1. Introduction

Heavy metal pollution is rapidly increasing, which induce many serious problems to the environment [1]. Excess heavy metals not only reduce soil fertility and crop yield, but also threaten human health via the food chain [2]. The essential micronutrient, Cu, serves crucial roles in a wide range of biological activities and redox reactions in plants [3,4]. However, excess Cu induces phytotoxicity in plants that may impair many physiological processes, including photosynthesis and pigment synthesis, oxidative stress, lipid peroxidation in membrane, and other metabolic disturbances [5,6]. Eventually, the activities related to tricarboxylic acid (TCA) cycle and PSII (photosystem II) reaction centers are drastically impaired [7].

A number of chelating agents, including ethylenediamine-tetraacetic acid (EDTA), N-(2-hydroxyethyl)-ethylenediaminetriacetic acid (HEDTA), and citric acid (CA), have an effective role in enhancing metal mobility and bioavailability, leading to enhanced phytoextraction [8,9,10]. However, CA, as a low molecular weight organic acid, plays an important role as an intermediate of the trichloroacetic acid (TCA) cycle, and improves the bioavailability and removal efficiency of heavy metals from contaminated soil [11]. The protective role of CA towards metal-induced oxidative stress has also captured scientists’ attention to increase pivotal enzymes’ activity in the antioxidant defense system [12,13,14].

Cu can induce reactive oxygen species (ROS) generation, which causes oxidative stress in plants [15]. To cope with metal toxicity, plants have evolved various response mechanisms. To this end, antioxidant defense systems are assumed to scavenge ROS under stress conditions in plants via increasing the ROS scavengers, such as free proline (Pro), peroxidases (PODs), superoxide dismutase (SOD), and catalase (CAT) [16,17].

In recent decades, metalloproteomic studies have been the focus of attention to elucidate the molecular mechanism against heavy metal toxicity in plants [18]. Therefore, the identification of stress-tolerant genes or proteins would be of great interest to decipher an effective molecular mechanism associated with metal stress tolerance in plants, including Brassica [19,20]. Although several studies associated with Cu tolerance have been conducted at the morphological and genetic level to identify the candidate genes and organic acid-assisted phytoextration in many plants, including Brassica [10,16,21,22,23], it is crucial to explore how CA regulates excess Cu in *B. napus* seedlings at the molecular level. Although transcriptomics offers many insights into deciphering numerous genes networks associated with heavy metal stresses, there still remains the mystery of the underlying molecular mechanism owing to the regulatory natures of genes at the transcriptional, translational, and post-translational level [24,25,26]. Proteomics, as a state-of-the-art technology, links these networks to protein products and addresses the biological function of proteins under various environmental stresses in crops [27]. Two-dimensional electrophoresis (2-DE) has been massively employed in the past two decades for studying protein abundance and its functional characterization on plants’ responses to heavy metal stresses, including Cd, Hg, Pb, and As [28,29,30,31,32]. However, several studies related to Cu stress have mostly been executed in *Triticum aestivum* L., *Oenothera glazioviana*, *Elsholtzia splendens*, *Phragmites australis*, and *Sorghum bicolor* L. [4,33,34,35,36]. In Brassica, several proteomics studies have been conducted till now to explore the molecular mechanism towards abiotic stresses [37,38,39,40,41,42,43,44,45,46,47]. The 2-DE method still has some drawbacks for the identification of low abundance and limited solubility proteins [47]. To overcome these problems, a label-free quantitative proteomic approach is introduced as a reliable and robust methodology that induces mass spectrometry stability and opens a new route to compare protein abundance by measuring peak intensities [48,49].

*Brassica napus* L., as a prominent candidate for phytoextaction, can accumulate large quantities of heavy metal in the shoots [10] and is highly feasible for decontamination of heavy metals from the soil [50,51]. Furthermore, application of exogenous CA promotes the metal uptake and translocation from roots to shoots, leading to boosting of the phytoextraction of metals, including Cu, and inducing tolerance against oxidative stress [10,16]. However, the response mechanisms of *B. napus* to Cu stress in combination with exogenous CA are unknown, especially at the protein level. Therefore, a label-free quantitative proteomic approach using an LTQ Orbitrap mass spectrometer was executed to analyze its morphological and biochemical responses coupled with Cu-responsive differentially abundant proteins (DAPs) in *B. napus* seedlings.

## 2. Results

### 2.1. Responses of Morphological, and Biochemical Responses to CA-Mediated Cu Stress in B. napus

**Figure 1:**
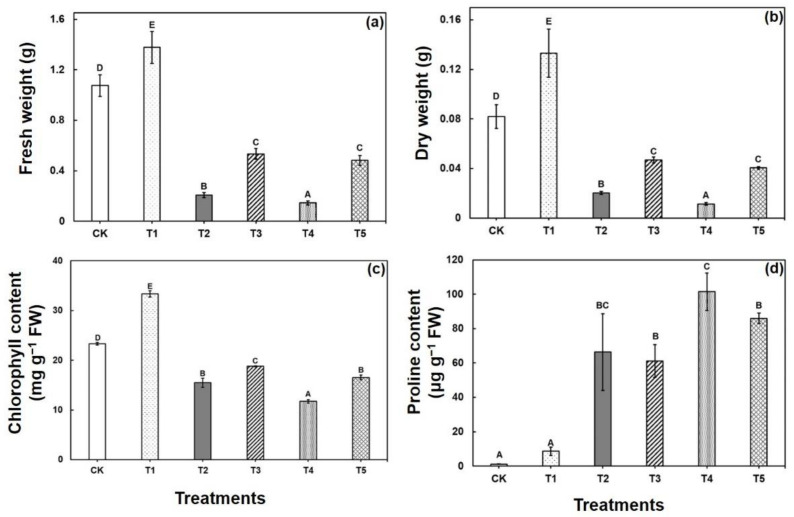
Effect of exogenous Cu and CA on fresh weight (**a**), dry weight (**b**), total chlorophyll (Chl) content (**c**), and proline (Pro) content (**d**) in Brassica seedlings with 0, 25, and 50 µM and 0 or 1.0 mM CA. Each value represents the mean of three replicates ±SE. Different letters indicate significant differences at *p* < 0.05 among treatments by Tukey’s test. The treatments CK, T1, T2, T3, T4, and T5 represent control, CA, Cu25 µM, CA + Cu25 µM, Cu50 µM, and CA + Cu50 µM respectively.

Brassica seedlings showed severe morphological disturbances after 7 days of Cu exposure. A considerable reduction in the fresh and dry weight was observed compared with the control (Figure 1). However, the most pronounced growth inhibition was observed in plants treated with the highest concentrations of CuSO_4_ (50 μM). The fresh and dry weight decreased significantly in response to Cu stress compared with control seedlings. However, the data represent that the highest amount of fresh weight and dry weight was found to be decreased drastically by 80% and 86%, respectively, under 50 μM CuSO_4_ as compared with the control (Figure 1a,b). Interestingly, addition of CA considerably ameliorated Cu toxicity in the *B. napus* by promoting plant fresh and dry weight in contrast to the stress treatments without CA.

Under Cu stress, chlorophyll (Chl) content decreased by 34% and 50% in the treated seedlings with increased copper concentration (25 μM and 50 μM), respectively, in 4 weeks harvested seedlings as compared with the control. Addition of CA along with Cu significantly increased total chlorophyll contents by 21% and 41% under Cu stress (25 μM and 50 μM, respectively) as compared with the respective Cu-treated plants without CA addition (Figure 1c). In leaves, Proline (Pro) content was increased by 55% and 84% in Cu-stressed seedlings (Cu25 and Cu50 μM, respectively) compared with the control. On the other hand, exogenous CA application noticeably decreased the elevated level of Pro in contrast to the stress treatments without CA (Figure 1d).

**Figure 2:**
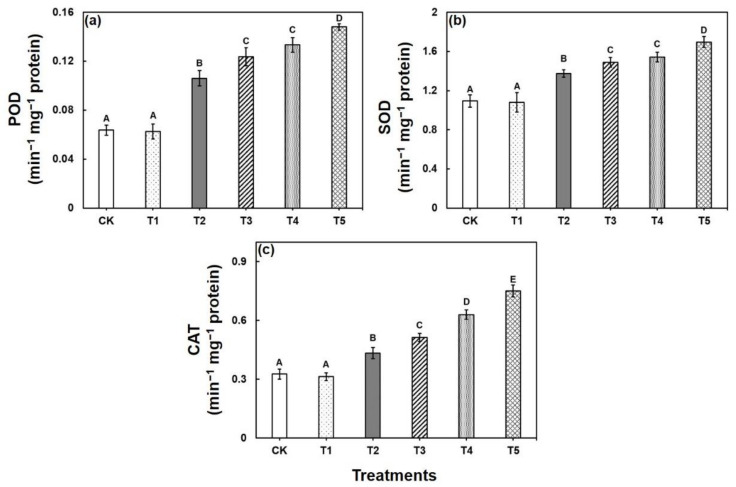
Effect of exogenous Cu and CA on enzyme activities (**a**) POD, (**b**) SOD, and (**c**) CAT in leaves of *B. napus* L. seedlings with 0, 25, and 50 µM and 0 or 1.0 mM CA. Each value represents the mean of three replicates ±SE. Different letters indicate significant differences at *p* < 0.05 among treatments by Tukey’s test.

Under Cu stress, POD activity increased significantly by 66% and 109% in both 25 µM and 50 µM, respectively, compared with the control (Figure 2). Addition of CA with Cu stress significantly increased POD activity further towards both concentrations of Cu in contrast to Cu stress alone (Figure 2). SOD activity showed substantial increase in Cu-stressed seedlings compared with the control plants. On the other hand, SOD activity further increased by 8 and 10%, when the Cu-stressed seedlings were exposed to CA (1.0 mM) compared with the Cu stress alone in Brassica seedlings. Compared with the control seedlings, CAT activity increased gradually with the increase in stress intensity. However, exogenous application of CA to the Cu-treated plants resulted in further improvement of CAT activity by 18 and 19% in contrast to Cu stress alone in the plants under 25 µM and 50 µM stress, respectively (Figure 2).

### 2.2. Changes of CA-Mediated Cu Stress on Proteome

**Figure 3:**
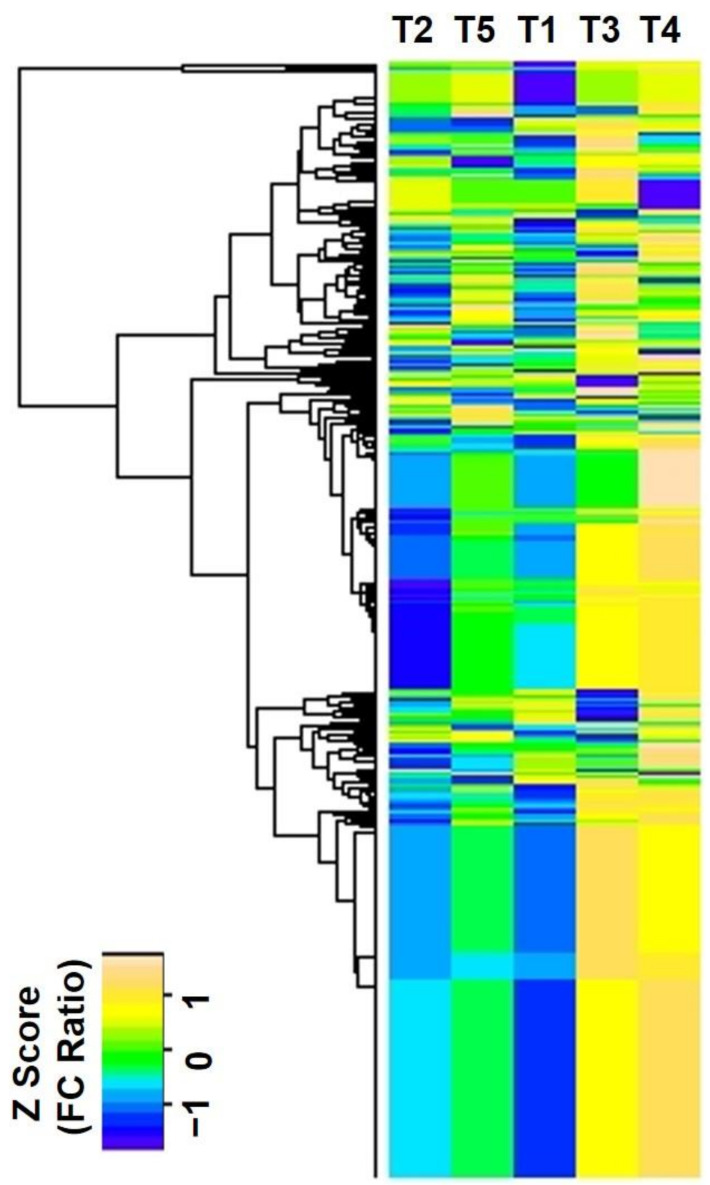
Heatmap analysis of the 426 differentially abundant proteins. Top, different treatments; left, protein tree. The color scale bar in the left, bottom corner indicates increased (yellow) and decreased (blue) levels of proteins in response to CA-mediated Cu stress.

Regarding dramatic alterations in the morphological and biochemical responses to CA-mediated Cu stress, we performed proteome analysis to explore the differential abundant proteins (DAPs) in the *B. napus* seedlings. The differential abundance profiles of the quantified proteins were displayed in a heatmap to compare the DAPs between different sample groups (Figure 3). The differential protein abundances were displayed by the ratio of fold changes from the normalized value of the exponentially modified protein abundance index (emPAI) as a heat map. Compared with control samples, four-hundred-and-twenty-six proteins were detected in all treatments, which are illustrated in the heat map based on their differential protein abundances (Figure 3).

Using an LTQ Orbitrap mass spectrometry, a total of 6345 proteins were identified in the leaves from all the treatment at a 95% confidence level. Among these 6345 proteins, a total of 1140, 1086, 1200, 990, 853, and 1076 proteins were identified in control, CA, Cu25 µM, CA + Cu25 µM, Cu50 µM, and CA + Cu50 µM treated samples in *B. napus* seedlings, whereas 552 proteins were commonly identified in all treatments (Figure S2).

**Figure 4:**
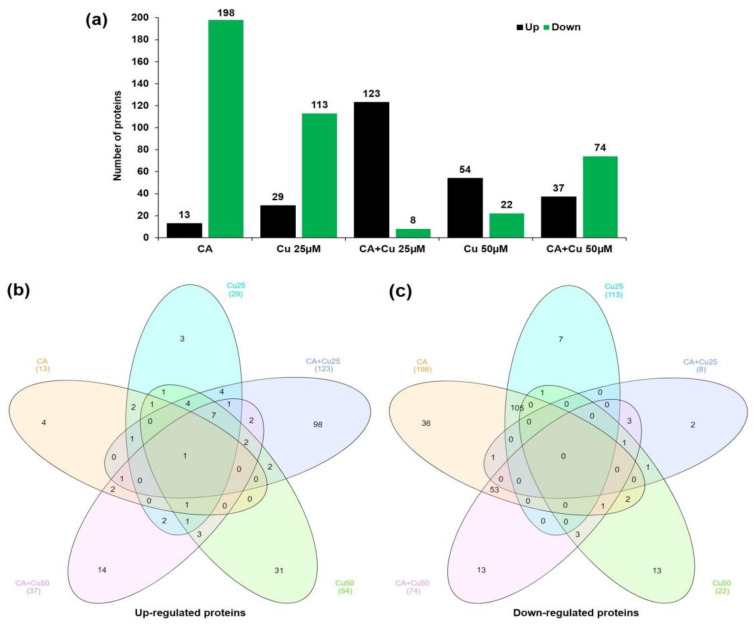
Identification and statistics analysis of DAPs under different treatment groups. (**a**) Number of up- or downregulated proteins between the CK group and different treatments. (**b**) Venn diagram analyses for upregulated proteins. (**c**) Venn diagram analysis for downregulated proteins.

Proteomic changes were first examined between the control and other treatment groups at different concentrations of CA and Cu. A total of 426 proteins were revealed to be differentially abundantly distributed across the different conditions in CA-mediated Cu stressed plants compared with control plants (Table S2). In the control versus CA comparison, a total of 211 DAPs, including 13 up-regulated and 198 down-regulated proteins, were identified; in the control versus Cu Cu25 µM comparison, 142 DAPs, including 29 up-regulated and 113 down-regulated proteins, were identified; in the control versus CA + Cu25 µM comparison, 131 DAPs, including 123 up-regulated and 8 down-regulated proteins, were identified; in the control versus Cu50 µM comparison, 76 DAPs, including 54 up-regulated and 22 down-regulated proteins, were identified; and in the control versus CA + Cu50 µM comparison, 111 DAPs, including 37 up-regulated and 74 down-regulated proteins, were identified (Figure 4a and Table S3). To infer the overlapping and unique proteins in various treatment groups, Venn diagrams of the DAPs representing up- and down-regulated proteins were analyzed (Figure 4b,c and Table S3). In particular, seven up-regulated DAPs were commonly detected in Cu25, CA + Cu25, Cu 50, and CA + Cu50 treatment groups, while a total of 105 down-regulated DAPs were identified between CA and Cu25 treatment groups. The higher number of up-regulated proteins (98 DAPs) in CA + Cu25 samples indicates the higher magnitude of proteins involved in response to CA-mediated Cu stress.

### 2.3. GO Analysis of the Total Identified Proteins in CA-Mediated Cu Stressed Plants

**Figure 5:**
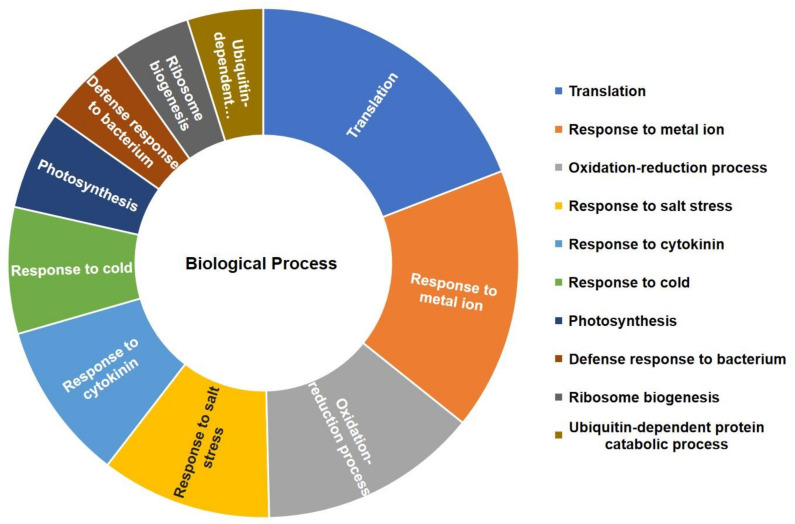
Gene ontology (GO) annotation (biological process) of the total identified proteins in *B. napus* seedlings in response to CA-mediated Cu stress using DAVID Bioinformatics.

DAVID functional annotation analysis revealed that the top GO-enriched identified proteins could be assigned to various functional GO terms (Figure 5). For the biological process categories, most of the identified proteins were involved in translation (131 proteins), response to metal ion (114 proteins), and glycolysis (95 proteins), respectively (Figure 5, Table S4); for the cellular component category, chloroplast (440 proteins), cytosol (341 proteins), and cytoplasm (259 proteins) exhibited the dominant GO terms (Table S4). ATP binding (146 proteins), structural constituents of ribosome (128 proteins), and protein binding (120 proteins) represented the most abundant groups in the molecular function category (Table S4). GO annotation analysis suggests that these top enriched GO terms play a vital role in plant response to Cu stress.

Using DAVID Bioinformatics, a total of 1256 proteins were mapped to the 31 KEGG pathways (Table S5). Of these, the topmost KEGG pathways were enriched at the metabolic pathways [(ath01100); 260, 15.84%], followed by biosynthesis of secondary metabolites [(ath01110); 147, 8.96%], ribosome [(ath03010); 123, 7.50%], biosynthesis of antibiotics [(ath01130); 106, 6.46%], and carbon metabolism [(ath01200); 88, 5.36%].

### 2.4. GO Analysis of the DAPs in CA-Mediated Cu Stressed Plants

To characterize the molecular functions and biological processes of the differentially abundant proteins in the various treatment groups involved in *B. napus* response to CA-mediated Cu stress, DAVID gene ontology (GO) enrichment-based clustering analyses were performed (Figures S3–S7, Table S6). Comparing the CA and CK groups, in the biological process category, 17 DAPs were related to response to metal ion, followed by response to stress (10) and oxidation-reduction process (10 DAPs). Chloroplast (39 DAPs), cytosol (28 DAPs), and chloroplast stroma (24 DAPs) showed the major categories in the cellular component. The significantly enriched molecular function GO terms were metal ion binding (14 DAPs), structural constituent of ribosome (7 DAPs), and chlorophyll binding (6 DAPs) (Figure S3). Comparing the Cu25 and CK groups, based on the biological category, the topmost GO enriched terms were related to response to metal ion (14 DAPs), followed by response to cytokinin (9 DAPs) and response to stress (8 DAPs). Based on cellular component, chloroplast (28 DAPs), cytosol (20 DAPs), and chloroplast stroma (19 DAPs) showed the dominant categories, whereas metal ion binding (9 DAPs), structural constituent of ribosome (7 DAPs), and proton-transporting ATP synthase activity (5 DAPs) represented the dominant categories regarding molecular function (Figure S4). Comparing the CA + Cu25 and CK groups, based on the biological category, the topmost GO-enriched terms were related to response to metal ion (13 DAPs), followed by oxidation-reduction process (7 DAPs) and photosynthesis (4 DAPs). Based on cellular component, chloroplast (31 DAPs), cytosol (26 DAPs), and chloroplast stroma (19 DAPs) showed the dominant categories. ATP binding (12 DAPs), copper ion binding (6 DAPs), and GTP binding (6 DAPs) represented the dominant categories regarding molecular function (Figure S5). Comparing the Cu50 and CK groups, based on the biological category, the topmost GO-enriched terms were related to response to metal ion (4 DAPs), followed by response to cytokinin (3 DAPs) and photorespiration (2 DAPs). Based on cellular component, chloroplast (11 DAPs), cytosol (8 DAPs), and chloroplast stroma (6 DAPs) showed the dominant categories. GTPase activity (2 DAPs) represented the dominant category regarding molecular function (Figure S6). Comparing the CA + Cu50 and CK groups, based on the biological category, the topmost GO-enriched terms were related to response to cold (7 DAPs), followed by oxidation-reduction process (6 DAPs) and photosynthesis (4 DAPs). Based on cellular component, chloroplast (21 DAPs), cytosol (17 DAPs), and plasma membrane (12 DAPs) showed the dominant categories. NAD binding (4 DAPs), copper ion binding (3 DAPs), and GAPDH activity (2 DAPs) represented the dominant categories regarding molecular function (Figure S7). The GO-enriched DAPs from the present study represented the most abundant categories, indicating that these categories were markedly regulated under Cu stress.

### 2.5. Potential Metabolic Pathways of the DAPs

**Figure 6:**
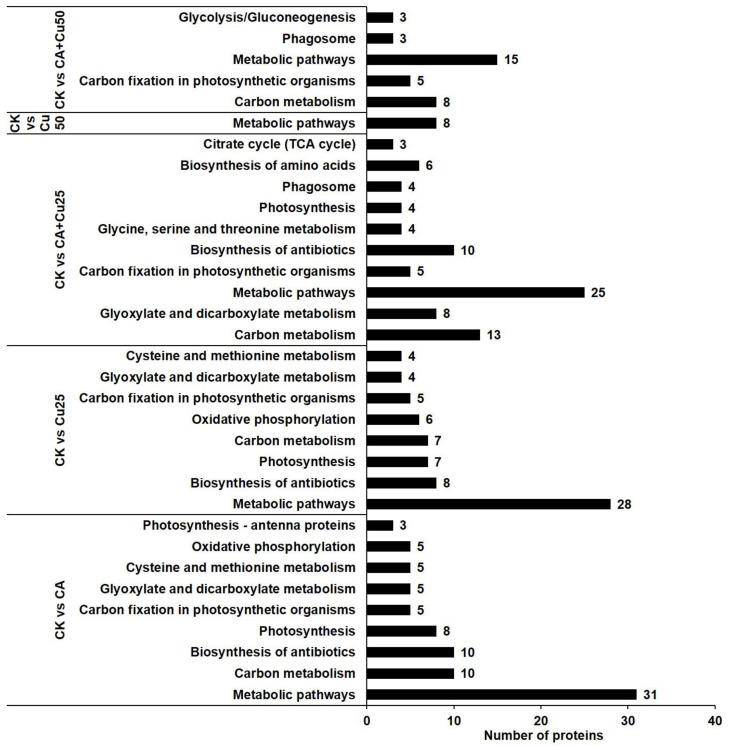
Top enriched KEGG pathways for DAPs identified in *B. napus* seedlings in response to CA-mediated Cu stress using DAVID Bioinformatics.

To gain insights into the metabolic changes, a potential metabolism pathway was constructed based on the DAPs altered by Cu stress in *B. napus* seedlings leaves using the KEGG database (http://www.kegg.jp/; access date, 6 October 2020). The top enriched KEGG pathways in the treatment groups are shown in Figure 6. Regarding all treatment groups, metabolic pathways (ath01100) was the most enriched, followed by carbon metabolism (ath01200), biosynthesis of antibiotics (ath01130), and carbon fixation in photosynthetic organisms (ath00710). The higher number of DAPs in CK and CA, CK, and CA + Cu25 samples represents the maximum number of proteins involved in alleviating Cu stress in *B. napus* (Table S7).

### 2.6. Validation of the Candidate Proteins by qRT-PCR

**Figure 7:**
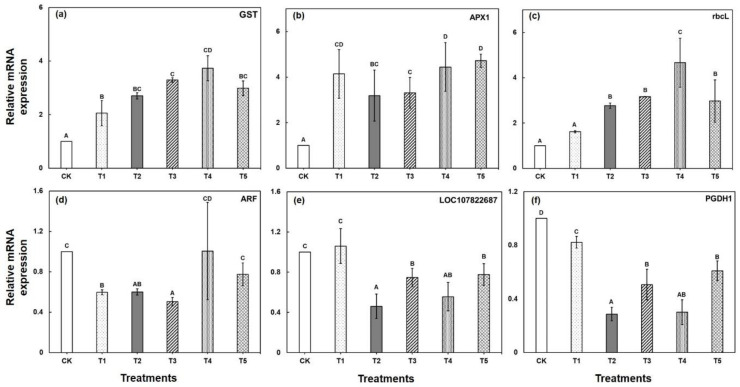
qRT-PCR validation of gene expression patterns in Brassica seedling leaves under CA-mediated Cu stress that correspond to six differentially abundant proteins. The subfigure (**a**–**c**) represents the up-regulated proteins corresponds to glutathione S-transferase U20, L-ascorbate peroxidase 1, and ribulose bisphosphate carboxylase large chain respectively; and (**d**–**f**) represents the down-regulated proteins corresponds to carbonic anhydrase, D-3-phosphoglycerate dehydrogenase 1, and ADP-ribosylation factor 2 respectively. Each value represents the mean of three replicates ±SE. Different letters indicate significant differences at *p* < 0.05 among treatments by Tukey’s test.

To confirm the accuracy of the DAPs and validate the correspondence between the transcript level of mRNA and protein abundance under CA-mediated Cu stress, a total of six candidate DAPs was selected for qPCR validation. The differential expression levels of four genes (GST, APX1, rbcL, LOC107822687) displayed a similar trend of change with the results of their corresponding protein abundance, suggesting that most of the proteins were regulated directly at the transcription level (Figure 7). Surprisingly, two genes, including PGDH1 and ARF, revealed an inconsistent relationship between the expression patterns of gene transcripts and protein abundance (Figure 7). This may be owing to a post-transcriptional modification of mRNAs. The qRT-PCR results revealed that the increased abundance of GST and APX1 could help Brassica plants to re-establish redox homeostasis under Cu stress. On the other hand, the exogenous application of CA enhanced the abundance level further in response to Cu stress.

## 3. Discussion

CA-mediated alleviation of Cu toxicity obviously may protect Brassica seedlings against excessive Cu. Prolonged Cu stress resulted in a drastic alteration in the overall morphological characteristics of Brassica seedlings, and modulated the Cu-responsive proteins abundance and the cell metabolism of plants (Figure 1, Table 1). However, CA application enhanced the growth parameters by maintaining photosynthetic membrane integrity and demonstrating the ameliorating action of CA in reducing Cu toxicity [52]. The morphological alterations coupled with the increased of DAPs of Cu-stressed Brassica seedlings (Figure 1, Table 1) are also observed in *Brassica napus*, *O. glazioviana*, and *Corchorus capsularis* [4,10,53], which suggests the protecting role of CA against Cu stress conditions.

Cu stress induced a high increase in the Pro level, whereas CA application reduced the accumulated Pro level in the Cu-stressed seedlings, improved osmotic adjustment of the plants, and confirmed the amelioration of stress (Figure 1d). Proline, as an osmoprotectant, plays a crucial role against osmotic disturbance, and is involved in various metabolic activities and improves stress tolerance in plant cells caused by various abiotic stresses, including Cu stress [54]. A previous report postulated that microorganisms provide crucial insights into the cellular defenses and stress response mechanisms as they increase the abiotic stress tolerance capacity of plants via amelioration of the oxidative damage and enhancing the content of photosynthetic pigments and and osmoprotective solutes [55,56]. Our findings were also found in earlier studies on heavy metal stress [57], including Cu stress [58]. The imbalance between the production of ROS and antioxidant defense induces oxidative stress in plants when exposed to environmental stress. Thereby, the major enzymatic antioxidants, including SOD, CAT, and POD, constitute the major enzymatic network that minimizes the ROS-induced oxidative stress [59]. In this study, antioxidant activity showed a pronounced increase under Cu stress. However, CA application resulted in a further increase in antioxidant activity of Cu exposure, which implied that exogenous CA provided a safeguard against ROS. A similar phenomenon was found in Brassica under heavy metal stress [16,60], which supports our findings.

Using the gel-free quantification method, we found that the abundance of 44 DAPs significantly changed in Cu treated seedlings compared with the control. However, the DAPs obtained from the present study were mainly associated in a variety of biological processes, including energy and carbohydrate metabolism (11 DAPs), CO_2_ assimilation and photosynthesis (13 DAPs), signal transduction and protein metabolism (6 DAPs), stress- and defense-related proteins (4 DAPs), sulfur assimilation and heavy metal detoxification (4 DAPs), cytoskeleton and cell wall-related proteins (3 DAPs), and development (3 DAPs) (Table 1).

### 3.1. Cu Stress Altered Energy and Carbohydrate Metabolism

In plants, carbohydrate and energy metabolisms represent vital biological processes for the maintenance of metabolic pathways and carbohydrate balance. The relationship between the accumulation of carbohydrates and enhanced HM tolerance has been reported in some plant species [61,62]. In the present study, a total of 11 DAPs were involved in the carbohydrate metabolism and CA cycle; of these, succinate—CoA ligase [ADP-forming] subunit beta (O82662), NADH dehydrogenase [ubiquinone] iron-sulfur protein 3 (P80261), two malate dehydrogenases (P57106, P93819), ATP synthase subunit beta-1 (P83483), and ATP synthase subunit alpha (A4QL04) showed increased abundance, while NADP-dependent glyceraldehyde-3-phosphate dehydrogenase (P93338), two fructose-bisphosphate aldolases (P16096, O65581), D-3-phosphoglycerate dehydrogenase 1 (O49485), and glyceraldehyde-3-phosphate dehydrogenase A (P12858) were decreased (Table 1). Under heavy metal stress, plants have developed complex and intricate regulatory network mechanisms to positively modulate the metabolic pathways of glycolysis and the TCA cycle [63].

Two DEPs involved in the TCA cycle pathway, including succinate—CoA ligase [ADP-forming] subunit beta (O82662), exhibited increased abundance under Cu stress, whereas D-3-phosphoglycerate dehydrogenase 1 (O49485) exhibited decreased abundance under a low concentration of Cu. Interestingly, however, exogenous CA treatment promoted the alleviation of Cu stress symptoms. A previous proteomic study in *O. glazioviana* subjected to Cu stress revealed over expression of succinate—CoA ligase [ADP-forming] subunit beta [4].

Moreover, two malate dehydrogenases (P57106, P93819), ATP synthase subunit beta-1 (P83483), and ATP synthase subunit alpha (A4QL04), considered as vital enzymes playing a crucial role in the TCA cycle pathway, were significantly regulated by Cu stress, which is in contrast to the results obtained from the previous studies under Cu stress in *Triticum aestivum* L. and *O. glazioviana* [4,34]. These highly abundant proteins provide potential clues to meet the important energy demand by maintaining their essential respiration and provide more glycolytically generated ATP by reinforcing the CA cycle and regulating the carbohydrate metabolism and energy production in Brassica plants under Cu stress.

### 3.2. Cu Stress Impairs the CO_2_ Assimilation and Photosynthesis

Heavy metal stress hinders the photosynthetic apparatus, leading to impairment of the most crucial biological process in plants. Decreased abundance of the photosynthesis-related proteins involved in the Calvin cycle and the photosynthetic electron transport chain were reported in plants, including Brassica subjected to heavy metals [34,64].

A total of 13 DAPs involved in CO_2_ assimilation and photosynthesis were identified in this study, of which photosystem II protein D1 (P36491), photosystem II protein D1 (P69561), photosystem II CP43 reaction center protein (A0ZZ31), photosystem I P700 chlorophyll a apoprotein A2 (A4QJB4), cytochrome b6 (A1EA38), chlorophyll a-b binding protein 6 (Q01667), chlorophyll a-b binding protein 4 (P27521), carbonic anhydrase (P27141), phosphoglycerate kinase (P29409), phosphoenolpyruvate carboxylase (P51062), and oxygen-evolving enhancer protein 1-1 (P23321) were reduced, while ribulose bisphosphate carboxylase large chain (A1E9T2) and photosystem II CP43 reaction center protein (A0A331) were induced under CA-mediated Cu stress (Table 1). Most of the (11 DAPs) photosynthesis-related protein species were significantly decreased in CA-mediated Cu-stressed Brassica seedlings. The decreased abundance of these proteins has an influence role on the D1 protein abundance in response to Cu stress that eventually hampered the overall stability of PSII [65]. The downregulation of these DAPs could be associated with the lower amount of photosynthetic pigment described above (Figure 1d) and the impaired photosynthetic machineries reported in the earlier studies [66]. On the other hand, two DAPs, named RuBisCo (A1E9T2) and PS II CP43 reaction center protein (A0A331), involved in regulating carbon fixation were significantly induced under Cu stress. The latter results agree with those obtained in Poplar that showed an increase in photosynthesis [67]. The obtained results suggest that enhanced carbon fixation has a potential role in modulating the energy production and carbohydrate metabolism in *B. napus* under Cu stress.

However, further increased abundance of these DAPs following CA treatment in Cu-stressed seedlings restored photosynthetic damage that may contribute to the tolerance to Cu-mediated oxidative stress.

### 3.3. Cu Stress Induces Changes in Signal Transduction and Protein Metabolism

The signal transduction plays a crucial role in regulating the gene expression of plants response to heavy metal stress [22,33]. In this proteome study, seven DAPs were identified involved in protein metabolism; of these, peptidyl-prolyl cis-trans isomerase CYP20-3 (P34791), GTP-binding nuclear protein Ran2 (P38547), GTP-binding nuclear protein Ran1 (P38546), chaperonin CPN60 (P35480), and serine hydroxymethyltransferase 4 (O23254) were induced, while 50S ribosomal protein L12-1 (P36210) and nucleoside diphosphate kinase III (O49203) were reduced under Cu stress (Table 1). GTP-binding proteins are well-known for molecular switches that provide crucial insights into plant signal transduction pathways, which participate in plant defense responses via producing plant secondary metabolites [68]. In a previous study, GTP-binding proteins were identified in *Phanerochaete chrysosporium* and *Elsholtzia splendens* rice by Cu treatment [33,69]. In *O. glazioviana*, two small GTP-binding proteins and Ras-related proteins showed increased abundance under Cu stress, which plays a critical role in signaling, cellular development, and cell cycle regulation [4]. Increasing evidence of these proteins indicates that the regulatory networks associated with the signal transduction pathway may be unraveled, induced by Cu toxicity. On the contrary, down-regulation of 50S ribosomal protein was identified in this study, leading to impaired protein metabolism, also reported in soybean earlier under short-term salt stress [70]. However, application of exogenous CA notably re-established this inhibitory effect caused by Cu stress.

### 3.4. Cu Stress Induces Stress-and Defense-Related Proteins

Plants have evolved a wide range of strategies to cope up with the heavy metal stress via stimulating the antioxidant enzymes, including SOD, CAT, and POD, which eventually enhanced the abundance of stress-and defense-related proteins [38].

Here, we identified four DAPs, including superoxide dismutase [Cu-Zn] 2 (O78310), superoxide dismutase [Cu-Zn] (P09678), stromal 70 kDa heat shock-related protein (Q02028), and L-ascorbate peroxidase 1 (Q05431), involved in stress and defense (Table 1). Notably, most of the stress- and defense-related proteins showed significant abundance under Cu25 and Cu50 treatment that could protect Cu-induced cell membranes from lipid peroxidation. The increased abundance of these proteins was also reported under Cu stress conditions in *O. glazioviana* and *Oryza sativa* L. [4,71]. Under CA treatment, the abundance of these DAPs showed a further increase after 7 days of Cu exposure. Collectively, these DAPs involved in stress response might help Brassica plants to scavenge ROS and maintain redox homeostasis under Cu stress.

### 3.5. Proteins Participate in Sulfur Assimilation and Heavy Metal Detoxification

Sulfur assimilation and GSH metabolism play crucial roles in regulating plant tolerance towards heavy metals [72]. In this study, a total of four sulfur assimilation and heavy metal detoxification related proteins, including cysteine synthase (P47999), glutathione S-transferase U20 (Q8L7C9), glutamine synthetase (Q42624), and 5-methyltetrahydropteroyltriglutamate—homocysteine methyltransferase 1 (O50008) were identified (Table 1). The increased abundance of cysteine synthase indicated an enhanced need for increasing the chelation processes through synthesis of glutathione (GSH), the predominant non-protein thiol, which plays an important role in plant stress responses that were observed in rice [71] and brown alga [73]. On the other hand, CA application further increased the abundance of these DAPs, which in turn enhanced the detoxification of ROS and, thereby, reduced oxidative damage in the Cu-exposed seedlings. The findings suggest that exogenous application of CA could be an effective approach to strengthen plant capacity to cope with the deleterious effects of heavy metal stress.

### 3.6. Proteins Related to Cytoskeleton and Cell Wall-Related Proteins

Cytoskeletal and cell wall-related proteins modulate rapidly when plants are subjected to heavy metal stress [5,74]. Three DAPs, including germin-like protein 1 (P45854), tubulin alpha-3 chain (O22349), and glucan endo-1,3-beta-glucosidase (P49236), involved in cytoskeleton and cell wall were identified in this study (Table 1). Germin-like protein (GLP) is considered as a dynamic plant glycoprotein that belongs to the cupin super family, and showed increased abundance in the present study. As a histidine-containing motif, GLP helps in binding metal ions and extracellular detoxification of Cu induced by oxidative stress [33,75].

### 3.7. Development

In this study, three DAPs, including ADP-ribosylation factor 2 (P51823), ADP-ribosylation factor 1 (P51821), and ADP-ribosylation factor (P49076), were remarkably down-regulated and are involved in plant growth and development (Table 1). The reduced abundance of the ADP-ribosylation factor resulted in a decrease in the overall growth of Brassica seedlings, leading to inhibited growth characteristics, which is consistent with the previous study [4].

## 4. Materials and Methods

### 4.1. Plant Materials, Growing Conditions, and Stress Treatments

Seeds of *B. napus* L. (cv. Jungmo 7001) were collected from Mokpo Experiment Station, National Institute of Crop Science, Rural Development Administration (RDA), Muan, Korea. The seeds were thoroughly washed with distilled water after sterilizing with 1% NaOCl for 20 min [76]. Then, seeds were washed with sterile deionized water and grown in controlled conditions (16 h day at 25 °C and 8 h night at 23 °C, 65 ± 5% relative humidity, 150 µmol·m^−2^·s^−1^ light intensity) for 5 days. Following germination, the healthy seedlings were transferred to plastic boxes and grown hydroponically for 21 days containing Hoagland solution [77]. The Hoagland solution was constantly aerated with air pump and renewed three times every week. After 21 days, the seedlings were exposed CuSO_4_ and citric acid (CA) for 7 days in the following manner: CA (1.0 mM) as T1, Cu (25 µM) as T2, Cu (25 µM) + CA (1.0 mM) as T3, Cu (50 µM) as T4, and Cu (50 µM) + CA (1.0 mM) as T5, following a completely randomized design (CRD) with three replications, whereas no Cu and CA was added to the control treatment (CK). Leaves were collected immediately from the control and treated seedlings, rinsed with de-ionized water, rapidly frozen in liquid nitrogen, and stored at −80 °C for the measurement of biochemical and proteome analyses. The overview of the experimental setup is illustrated in Figure S1 to investigate the CA-assisted Cu tolerance in Brassica seedlings.

### 4.2. Growth Parameters

Shoot fresh weights (g) and root fresh weights (g) were measured from each of the collected samples. For the measurement of dry weight of the shoot and root (g), the samples were dried in a force oven at 65 °C for 72 h [78].

### 4.3. Measurement of Biochemical Parameters

Chlorophyll contents of the leaf extract were determined as previously described with minor modification [79]. In brief, fresh leaves (0.2 g) of control and each treatment were cut to small size and soaked in 80% acetone (10 mL) for 2 h at room temperature (RT) and homogenized the samples with mortar and pestle. The homogenate was centrifuged at 12,000× *g* for 10 min at RT. The final volume of the filtrate was maintained at 10 mL. The pigments were extracted, and the absorbance was measured at 645 and 663 nm on a UV/vis spectrophotometer. The chlorophyll contents were calculated by the following formula:

Chlorophyll a = [12.7(D663) − 2.69(D645)] × [v/1000 × w]

Chlorophyll b = [22.9(D645) − 4.68(D663)] × [v/1000 × w]

Total chlorophyll = Chl a + Chl b

The Proline (Pro) content was estimated according to the method as described previously with minor modifications [80]. Fresh leaf samples (0.5 g) were homogenized in 3% aqueous sulfosalicylic acid and the resultant homogenate was centrifuged at 11,500× *g* for 15 min. Supernatant (2 mL) was mixed with equal volume of glacial acetic acid (2 mL) and ninhydrin (2 mL). The resultant mixture was boiled at 100 °C for 1 h and then separated with 4 mL of toluene, and the absorbance of the chromophore was measured at 520 nm with toluene as blank. Pro concentration was calculated using a calibration curve developed with Pro standard. The proline content was expressed as the unit’s μg per gram-fresh weight (μg g^−1^ FW).

### 4.4. Estimation of Antioxidant Enzyme Activities

The antioxidant enzyme activities were determined using a UV-spectrophotometer. The fresh leaf samples (0.5 g) were homogenized with 50 mM of potassium phosphate buffer, 1% (*w*/*v*) polyvinylpyrrolidone (pH 7.8), followed by incubation at 40 °C for 10 min. The homogenate was then centrifuged at 4000× *g* for 15 min at 4 °C and the extract was used for the measurement of POD and SOD activity [81]. Briefly, POD activity was measured by adding 1.5 mL of 0.5 M pyrogallol and 0.5 mL 1% hydrogen peroxide to 0.5 mL plant extract. The reaction mixture was incubated at 25 °C and the absorbance was measured at 420 nm at 30 s interval for 30 min. SOD activity was measured by adding 4 mL of reaction mixture containing 63 μM nitroblue tetrazolium (NBT), 13 mM L-methionine, 0.1 mM EDTA, 13 μM riboflavin, and 0.05 M sodium carbonate to 0.5 mL enzyme extract. The reaction tubes were kept under 15 W fluorescent lamps for 15 min and then incubated in the dark for another 15 min. The absorbance was recorded at 560 nm and 1 U of SOD represented the amount that inhibited NBT photoreduction by 50% at 25 °C. The activity of CAT was measured using a hydrogen peroxide assay. Enzyme extract (0.2 mL) was mixed in 1 mL of reaction mixture (containing 65 mM hydrogen peroxide in 60 mM sodium phosphate buffer) and incubated at RT for 4 min. The reaction was stopped by using 1 mL of 32.4 mM of ammonium molybdate and the yellow complex was measured at 405 nm [82].

### 4.5. Protein Extraction

The procedure for protein extraction from the leaves of Brassica seedlings was performed using the trichloroacetic acid (TCA)/acetone precipitation method, as previously described [35] with some modifications. Frozen leaves tissues (0.5 g) were ground with a mortar and pestle in liquid nitrogen. The resulting powder was homogenized in 10 mL of ice-cold 10% TCA in acetone with 0.07% (*v*/*v*) 2-mercaptoethanol (2-ME). The homogenate was sonicated for 10 min, followed by incubation at −20 °C for 1 h. The precipitate was then collected by centrifugation at 9000× *g* at 4 °C for 20 min. Afterwards, the pellet was washed several times with ice-cold acetone (containing 0.07% (*v*/*v*) 2-ME) and centrifuged again at 4 °C. The samples were vacuum-dried with a Speed-Vac (Hanil Science Medical, Modulspin 31, Seoul, South Korea) for 10 min. The dried sample was then suspended in a “lysis buffer (8 M urea, 2 M thiourea, 5% CHAPS, and 2 mM tributylphosphine)”. After “incubation” for 1 h at room temperature, the suspension was centrifugated at 20,000× *g* for 20 min at 25 °C, and the protein-containing supernatant was collected. Finally, the protein content was measured according to the method of Bradford (Bio-Rad) using bovine serum albumin (BSA) in lysis buffer as the standard [83].

### 4.6. Purification, Digestion of Extracted Proteins

The extracted proteins (150 μg) from Brassica leaves were purified using the methanol-chloroform method as previously described [84]. Briefly, after adjusting the sample volume to 150 μL, 600 μL methanol was added and the sample was mixed by vortexing; then, 150 μL chloroform was added and the sample was mixed again by vortexing. To induce phase separation, 450 μL of H_2_O was added to the sample, which was then mixed by vortexing. The solution was centrifuged at 20,000× *g* for 10 min at RT. The upper aqueous phase was carefully discarded, and 450 μL methanol was then added slowly to the remaining organic phase. The samples were centrifuged at 20,000× *g* for 10 min at RT, the supernatant was removed, and the pellet was air-dried for 10 min and resuspended in 50 mM NH_4_CO_3_. Each sample was reduced with 50 mM dithiothreitol for 30 min at 56 °C and then alkylated with 30 mM iodoacetamide for 30 min at 37 °C in the dark. The alkylated proteins were digested with trypsin (Promega, Madison, Wi, USA) at a for 16 h at 37 °C. The peptides were eluted into a clean tube and stored at 4 °C prior to tandem mass spectrometry (MS/MS) analysis.

### 4.7. LC-MS/MS Analysis

The extracted tryptic peptides were analyzed using an LTQ Orbitrap mass spectrometer (Thermo Fisher, Bremen, Germany) coupled with an 1100 nano-flow HPLC system (Agilent) by a nano electrospray ion source. A two-column setup was used. The pre-column, waste line, and analytical column (C18 AQ, 3 µm, 100 µm × 15 cm, Sciex Nano LC, Framingham, MA, USA) were interconnected using a three-way tee connector. An auto sampler was used to load aliquots (10 µL) of the peptide solutions onto a C18 trap column (Accaim Pep Map 100, 75 µm × 2 cm, nano Viper C18, 3 µm, Thermo Fisher Scientific). The mobile phases, A and B, were composed of 0 and 100% acetonitrile, respectively, each containing formic acid (0.1%). The peptides were desalted and concentrated on the trap column for 10 min at a flow rate of 10 µl/min with buffer A (0.1% formic acid) and eluted with a 150 min linear gradient from 0 to 50% buffer B (100% acetonitrile, 0.1% formic acid) at a flow rate of 300 nL/min. After the gradient, the column was washed with at least 10 column volumes of 100% solvent B in order to avoid sample carryover, and re-equilibrate with buffer A. All MS/MS spectra were acquired in a data-dependent mode for fragmentation of the five most abundant peaks from the full MS scan with 35% normalized collision energy. The separated peptide ions eluted from the analytical column were entered into the mass spectrometer at an electrospray voltage of 2.2 kV. The dynamic exclusion duration was set at 180 s and exclusion mass width 0.5 Da. MS spectra were acquired with a mass range of 150–2000 *m*/*z*.

### 4.8. Database Search and Protein Quantification

The obtained MS/MS spectra were searched against universal Protein Resource database (green plant with UniProt release 2018_10, 59345 entries, http://www.uniprot.org; access date, 8 January 2020) with Mascot Daemon (Version 2.4.1, Matrix Science, London, UK). Peptides were identified with monoisotopic mass selected, a precursor mass tolerance of ±1.5 Da, a fragment mass tolerance of ±0.8 Da, two missed trypsin cleavage, and fixed modification of carbamidomethyl cysteine. The threshold score/expectation value for accepting individual spectra was based on Mascot ion score threshold (0.05) as the standard ion score threshold specifically calculated by Mascot for each database search. As an indication of identification certainty, the false discovery rates for peptide and protein matches above identity threshold were calculated by Peptide Validator at 1.0%. The peptide score is −10 × Log (P), where P is the probability that the observed match is a random event. Individual peptide scores were considered as identity or extensive homology (*p* < 0.01).

Label-free quantitation analysis was performed according to the previously reported protocols [85]. Common contaminants and reverse decoy matches were removed from the protein identification list. At least two unique peptides per protein were required for a protein identification. Only proteins that were identified and quantifiable in at least two technical of at least four biological replicates in each group were used for relative quantification. The arithmetic mean was used to obtain the average label-free quantification intensity within each biological group. For statistical evaluation, a two-sided t-test was used. The *p*-value was corrected using false discovery rate (FDR)-based multiple hypothesis testing. Both t-test and FDR-based multiple hypothesis testing were carried out with the default settings of the Perseus statistics software. All data were normalized using a linear regression analysis.

### 4.9. Bioinformatics Analysis of the Identified Proteins

The identified proteins were categorized based on gene ontology classification such as biological processes, molecular activity, and cellular components using DAVID Bioinformatics Tools (https://david.ncifcrf.gov/; access date, 6 October 2020). Protein abundances were visualized as a heat map, and hierarchical clustering was applied with complete linkage based on Pearson distance metrics. The heat map was generated using the heatmap3 package of the R software (R-3.5.2, www.r-project.org; access date, 14 October 2020). Fold changes of the differentially abundant proteins were calculated by comparing the abundances among the treated and control samples. The Kyoto Encyclopedia of Genes and Genomes (KEGG) database (http://www.genome.jp/kegg/pathway.html; access date, 16 October 2020) was employed to deepen the knowledge of various pathways and their involvement in oxidative stress.

### 4.10. RNA Isolation, cDNA Synthesis, and Gene Expression Analysis by qRT-PCR

Fresh leaves (0.5 g) were ground using a mortar and pestle on liquid N and transferred to the 2.0 mL micro centrifuge tube. The samples were vortexed with 1 mL of RNA iso plus (Takara Bio Inc. Shiga, Japan). The samples were centrifuged at 12,000 rpm for 15 min at 4 °C. After incubation for 5 min at RT, the samples were transferred to a new 1.5 mL tube, and 200 μL of chloroform was added and it was shaken rigorously for 15 s. The samples were then centrifuged once more at the same condition. Later, 500 μL iso-propanol was added into the sample and transferred to a new 1.5 mL tube. The samples were incubated for 2 min at RT while being gently inverted. After incubating, the samples were centrifuged at the same condition one more time. Pellet remained in the tube, while supernatant was removed. The pellet was centrifuged with 75% of 1 mL ethanol at 8000 rpm for a few min at 4 °C and the ethanol was removed from the tube. This cleaning step was repeated several times. The samples were air-dried briefly for 3 to 5 min. The mRNA concentration was measured using nano drop-1000 (NanoDrop Technologies, Inc., Wilmington, DE, USA). The concentration of RNA was considered as ≥200 ng/μL for subsequent analysis. The cDNA synthesis was performed with 1 μg of total RNA using cDNA synthesis kit (Bio-Rad, Hercules, CA, USA), and qRT-PCR was performed using CFX96 Real-Time system (BIORAD, Hercules, CA, USA). The total reaction mixture (20 μL) containing 10 μL of iQTM SYBR^®^ Green Supermix, 2 μL of template cDNA, 0.8 μL of forward primer (10 μM), 0.8 μL of reverse primer (10 μM), and 6.4 μL of DEPC treated H_2_O was run in a CFX96 Real-Time system. The PCR system was programed in the following manner: 95 °C for 30 s, followed by 40 cycles at 95 °C for 5 s and 60 °C for 30 s. The relative gene expression was analyzed using the dd−∆Ct method [86], using BnaActin as an internal control. Three replicates per biological sample were maintained in each treatment for the qRT-PCR experiment. The designed primers for qRT-PCR analysis are shown in Table S1.

### 4.11. Statistical Analyses

Statistical differences were analyzed by analysis of variance (ANOVA) and Student’s t-test at a level of significance of *p* ≤ 0.05 between control and treated samples using SAS package, Version 9.4. All data are displayed as the mean ± standard error of the mean of at least three independent biological replications.

## 5. Conclusions

The quantitative proteomic analysis provides new insights into the detrimental effects of Cu stress on *B. napus* seedlings. Elevated concentrations of Cu impaired the morphological characteristics by inducing oxidative stress. Our study revealed that exogenous CA application alleviated the Cu toxicity by reducing oxidative stress and enhancing antioxidant enzyme activities, and plays a vital role in biochemical regulatory processes, particularly in protein modulation. Under CA treatment, DAPs showed an increased abundance, indicating a crucial role of CA in attenuating the adverse effects of Cu stress in *B. napus*. However, most of the DAPs are mainly associated with carbohydrates and energy metabolism, signal transduction and protein metabolism, stress and defense, photosynthesis, and citrate cycle (TCA cycle). The accuracy and authenticity of the proteomic data obtained from the present study were well confirmed using RT-qPCR analysis. The overall findings expand our knowledge and provide new clues for the molecular tolerance mechanism responsible for plant responses to Cu stress. Taken together, the present results pave the way for further detailed investigations at the field level to facilitate comprehensive clarification of molecular mechanisms involved in Cu stress responses and possible role of CA to revoke the negative effects of Cu toxicity in *B. napus*.

## Figures and Tables

**Table 1:** List of differentially abundant proteins (DAPs) identified in leaves of *B. napus* seedlings exposed to CA-mediated Cu stress for 7 days.

Accession Number	Protein Description	Gene Name	Coverage	Peptides	Fold Change				
					T1/CK	T2/CK	T3/CK	T4/CK	T5/CK
Energy and Carbohydrate Metabolism									
O82662	Succinate—CoA ligase [ADP-forming] subunit beta	At2g20420	15%	5	1.464	1.946	3.206	1.853	1.605
P93338	NADP-dependent glyceraldehyde-3-phosphate dehydrogenase	GAPN	12%	6	0.903	0.960	1.000	0.965	0.915
P80261	NADH dehydrogenase [ubiquinone] iron-sulfur protein 3	NAD9	21%	3	1.098	1.401	1.634	1.193	1.342
P57106	Malate dehydrogenase 2	MDH2	28%	10	0.942	1.023	1.324	1.039	1.170
P93819	Malate dehydrogenase 1	MDH1	37%	11	0.985	1.044	1.379	1.097	1.179
P16096	Fructose-bisphosphate aldolase	N/A	16%	6	0.774	0.774	0.950	0.950	0.802
O65581	Fructose-bisphosphate aldolase 5	FBA5	13%	3	0.673	0.501	0.830	0.602	0.776
P83483	ATP synthase subunit beta-1	At5g08670	48%	24	1.212	1.271	1.295	1.323	1.034
A4QL04	ATP synthase subunit alpha	atpA	29%	15	1.164	0.874	1.026	1.561	1.078
P12858	Glyceraldehyde-3-phosphate dehydrogenase A	GAPA	19%	14	0.892	0.850	0.985	1.035	0.963
O49485	D-3-phosphoglycerate dehydrogenase 1	PGDH1	14%	7	0.628	0.734	1.004	1.090	0.744
CO_2_ assimilation and photosynthesis									
P36491	Photosystem II protein D1	psbA	16%	6	0.692	0.744	1.233	0.904	0.793
P69561	Photosystem II protein D1	psbA	16%	6	0.692	0.744	1.099	0.789	0.793
A0A331	Photosystem II CP43 reaction center protein	psbC	21%	8	1.481	1.535	2.054	1.855	0.642
A0ZZ31	Photosystem II CP43 reaction center protein	psbC	18%	6	0.627	0.733	1.004	0.910	0.744
A4QJB4	Photosystem I P700 chlorophyll a apoprotein A2	psaB	13%	6	0.842	0.619	0.766	0.974	0.708
A1EA38	Cytochrome b6	petB	24%	4	0.872	0.854	0.901	0.863	0.903
Q01667	Chlorophyll a-b binding protein 6	LHCA1	19%	9	0.991	0.945	1.102	0.950	0.998
P27521	Chlorophyll a-b binding protein 4	LHCA4	17%	5	0.904	0.862	1.004	1.048	0.910
P27141	Carbonic anhydrase	LOC107822687	13%	4	0.657	0.783	1.004	0.860	0.738
A1E9T2	Ribulose bisphosphate carboxylase large chain	rbcL	19%	13	1.004	1.099	1.233	1.048	1.089
P29409	Phosphoglycerate kinase	N/A	29%	16	0.859	0.818	1.001	1.048	0.916
P51062	Phosphoenolpyruvate carboxylase	N/A	8%	7	1.211	1.110	0.775	0.731	0.673
P23321	Oxygen-evolving enhancer protein 1-1	PSBO1	47%	19	0.911	0.709	1.001	1.055	0.899
Signal transduction and Protein Metabolism									
P34791	Peptidyl-prolyl cis-trans isomerase CYP20-3	CYP20-3	27%	5	1.004	0.907	1.004	1.130	0.987
P38547	GTP-binding nuclear protein Ran2	RAN2	18%	4	0.972	1.474	1.476	1.536	1.560
P38546	GTP-binding nuclear protein Ran1	RAN1	18%	4	0.972	1.474	0.759	1.536	0.414
P35480	Chaperonin CPN60, mitochondrial	LOC106437789	18%	10	1.786	1.823	2.514	1.699	1.614
P36210	50S ribosomal protein L12-1	RPL12A	16%	3	0.904	0.862	1.004	0.935	0.910
O49203	Nucleoside diphosphate kinase III	NDPK3	9%	3	0.904	0.862	1.004	1.048	0.910
Stress-and Defense-related proteins									
O78310	Superoxide dismutase [Cu-Zn] 2	CSD2	13%	6	1.950	0.628	1.476	1.536	1.560
P09678	Superoxide dismutase [Cu-Zn]	SODCC	18%	3	0.875	1.475	1.255	1.048	0.910
Q02028	Stromal 70 kDa heat shock-related protein	HSP70	10%	7	0.911	1.046	1.511	1.363	1.210
Q05431	L-ascorbate peroxidase 1	APX1	14%	4	1.096	1.753	1.186	2.735	1.038
Sulfur assimilation and heavy metal detoxification									
P47999	Cysteine synthase	OASB	20%	7	1.233	1.240	1.002	1.148	0.970
Q8L7C9	Glutathione S-transferase U20	GSTU20	9.70%	3	1.904	1.321	1.643	1.048	1.400
Q42624	Glutamine synthetase	GLN2	25%	14	0.941	0.952	1.008	1.252	1.176
O50008	5-methyltetrahydropteroyltriglutamate—homocysteine methyltransferase 1	MS1	29%	26	1.046	0.991	1.370	1.373	1.074
Cytoskeleton and cell wall-related proteins									
P49236	Glucan endo-1,3-beta-glucosidase	BGL	8%	3	1.111	0.821	3.215	9.134	1.093
P45854	Germin-like protein 1	GLP1	25%	5	1.093	1.042	1.215	1.268	1.101
O22349	Tubulin alpha-3 chain	TUBA3	14%	8	0.952	0.884	0.794	0.862	1.004
Development									
P51823	ADP-ribosylation factor 2	ARF	46%	7	0.404	0.379	1.273	0.085	1.302
P51821	ADP-ribosylation factor 1	ARF1	46%	7	0.239	0.240	0.929	0.052	0.235
P49076	ADP-ribosylation factor	ARF1	46%	7	0.703	0.463	0.109	0.109	0.419

## Data Availability

The mass spectrometry data have been deposited to the MassIVE consortium (http://massive.ucsd.edu/ProteoSAFe/static/massive.jsp; access date, 20 April 2021) with the dataset identifier MSV000087241.

## Supplementary Materials

The following are available online at https://www.mdpi.com/article/10.3390/ijms22115879/s1, Figure S1. Schematic representation of the experimental setup used to investigate the CA assisted Cu-tolerance in *B. napus* seedlings; Figure S2. Venn diagram of total identified proteins showing common and unique proteins in each comparison groups; Figure S3: significantly enriched GO terms for the DAPs between the treatment groups (CK vs. CA) in *B. napus* seedling leaves under CA-mediated Cu stress; Figure S4: significantly enriched GO terms for the DAPs between the treatment groups (CK vs. Cu25 µM) in *B. napus* seedling leaves under CA-mediated Cu stress; Figure S5: significantly enriched GO terms for the DAPs between the treatment groups (CK vs. CA + Cu25 µM) in *B. napus* seedling leaves under CA-mediated Cu stress; Figure S6: significantly enriched GO terms for the DAPs between the treatment groups (CK vs. Cu50 µM) in *B. napus* seedling leaves under CA-mediated Cu stress; Figure S7: significantly enriched GO terms for the DAPs between the treatment groups (CK vs. CA + Cu50 µM) in *B. napus* seedling leaves under CA-mediated Cu stress; Table S1: primers used for qRT-PCR analysis; Table S2: list of differentially abundant proteins (DAPs) identified in this study. Cells with blue and yellow color indicate significant fold changes of increase or decrease in response to CA-mediated Cu stress; Table S3: list of differentially abundant proteins among the treatment groups; Table S4: GO annotation of the total identified proteins in *B. napus* seedlings in response to CA-mediated Cu stress using DAVID Bioinformatics; Table S5: KEGG pathways of the total identified proteins in *B. napus* seedlings in response to CA-mediated Cu stress using DAVID Bioinformatics, Table S6: significantly enriched GO terms for the DAPs among the treatment groups in *B. napus* under CA-mediated Cu stress; Table S7: list of significantly enriched KEGG pathways for the DAPs among the treatment groups in *B. napus* seedlings in response to CA-mediated Cu stress using DAVID Bioinformatics.
