# Supplementary material for: Proteome Changes Reveal the Protective Roles of Exogenous Citric Acid in Alleviating Cu Toxicity in Brassica napus L
Source: Int J Mol Sci. 2021 May 30;22(11):5879. doi: 10.3390/ijms22115879 (PMC8198124; doi:10.3390/ijms22115879)
Supplement: Supplementary file 1 [file ijms-22-05879-s001.zip › ijms-1235003-SI/Supplementary materials/Supplementary figures.pdf]

Proteome Changes Reveal the Protective Roles of Exogenous CA in Alleviating Cu Toxicity in *Brassica napus* L.

Young-Hwan Ju [1], Swapan Kumar Roy [1, 2], Aritra Roy Choudhury [3], Soo Jeong Kwon [1], Ju-Young Choi [1], Md Atikur Rahman [4], Tomoyuki Katsube-Tanaka [5], Tatsuhiko Shiraiwa [5], Moon Soon Lee [7], Kun Cho [6*], and Sun-Hee Woo [1*]

* Correspondence: shwoo@chungbuk.ac.kr (S.H.W.) and chokun@kbsi.re.kr (K.C.); Tel: +82-43-261-2515

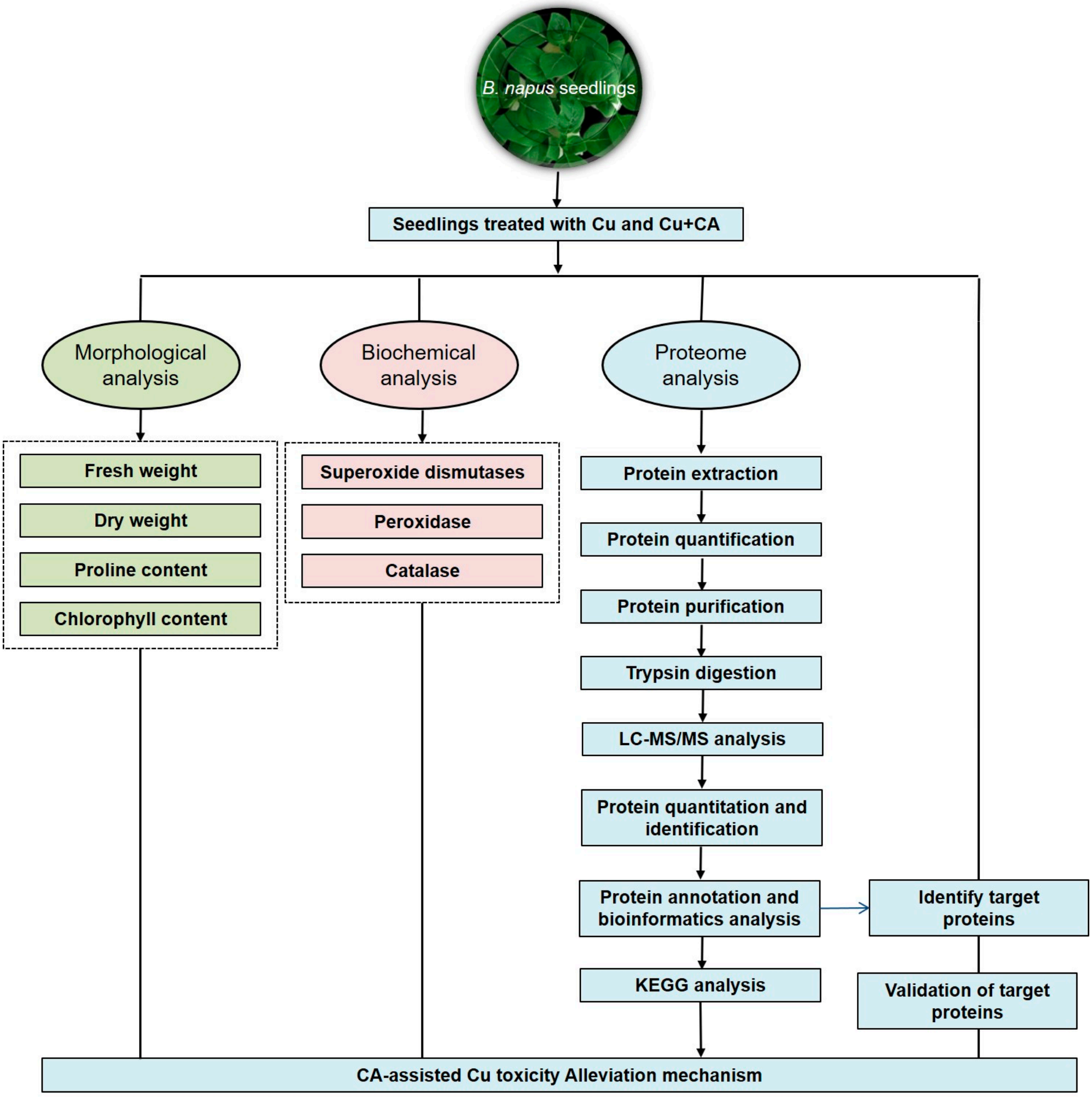

Figure S1. Schematic representation of the experimental setup used to investigate the CA assisted Cu-tolerance in B. napus seedlings

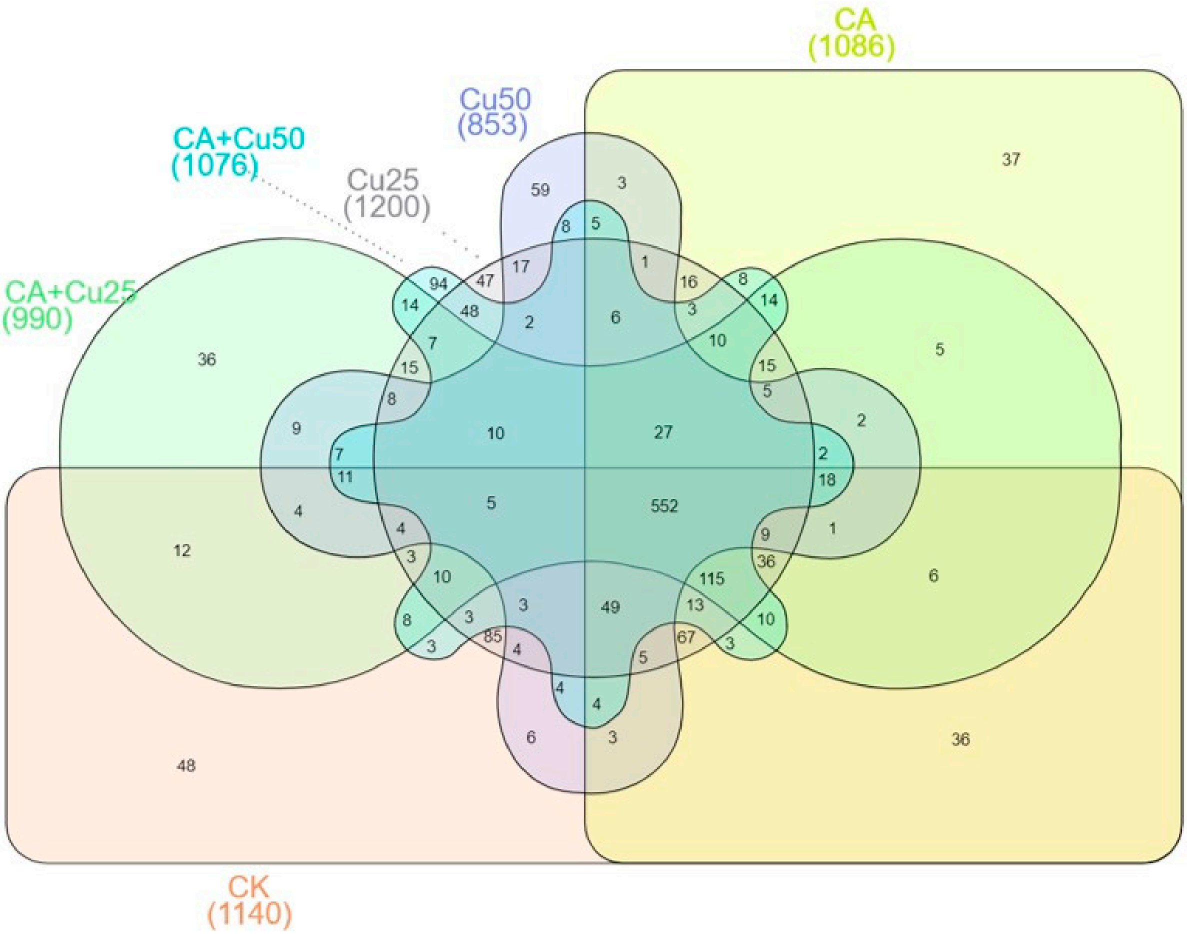

Figure S2. Venn diagram of total identified proteins showing common and unique proteins in each comparison groups.

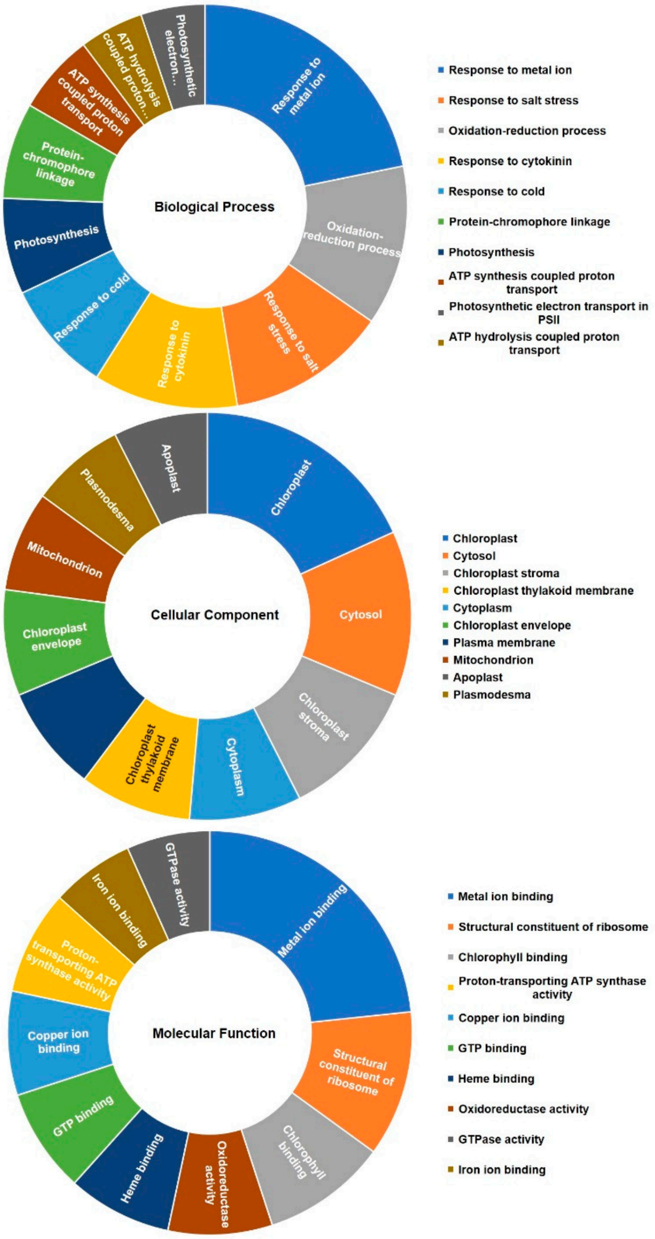

Figure S3. Significantly enriched GO terms for the DAPs between the treatment groups (CK Vs CA) in *B. napus* seedling leaves under CA-mediated Cu stress.

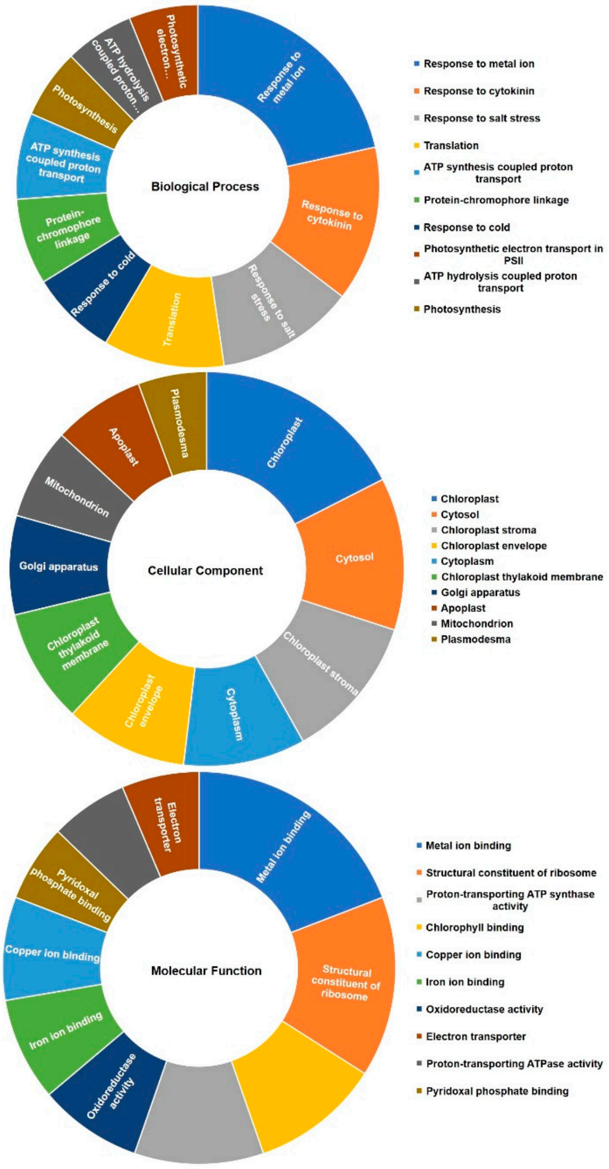

Figure S4. Significantly enriched GO terms for the DAPs between the treatment groups (CK Vs Cu25 μM) in *B. napus* seedling leaves under CA-mediated Cu stress.

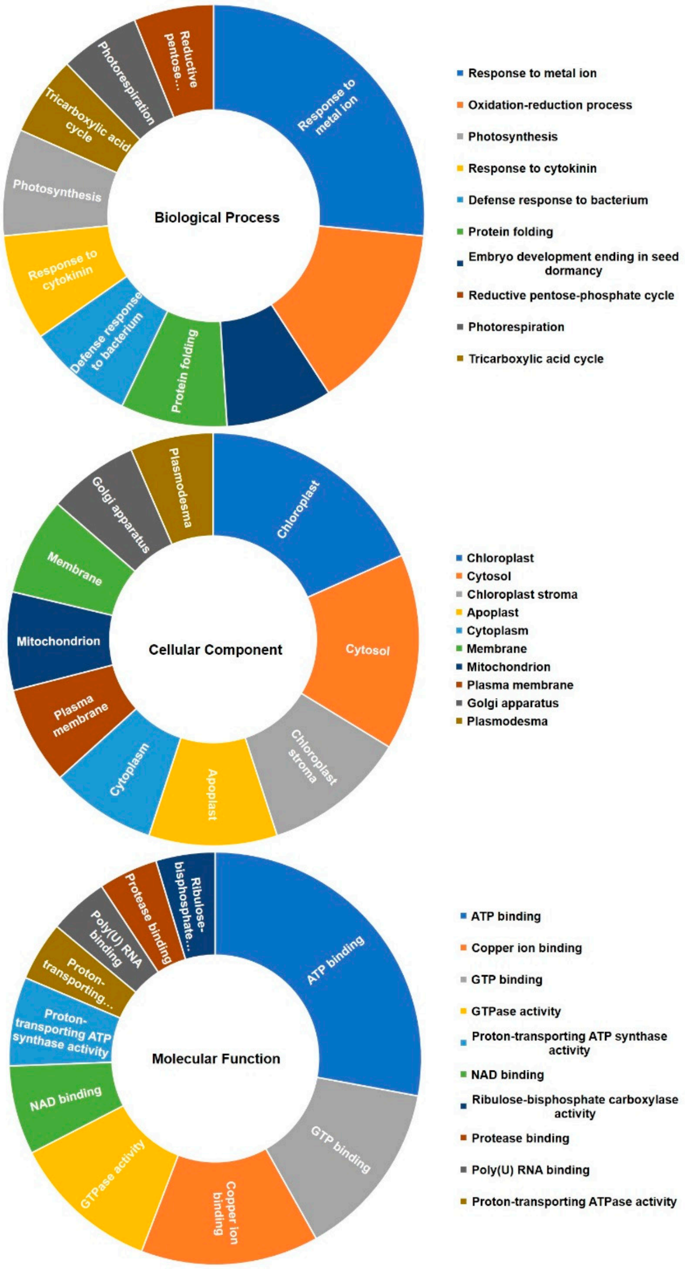

Figure S5. Significantly enriched GO terms for the DAPs between the treatment groups (CK Vs CA+Cu25 μM) in *B. napus* seedling leaves under CA-mediated Cu stress.

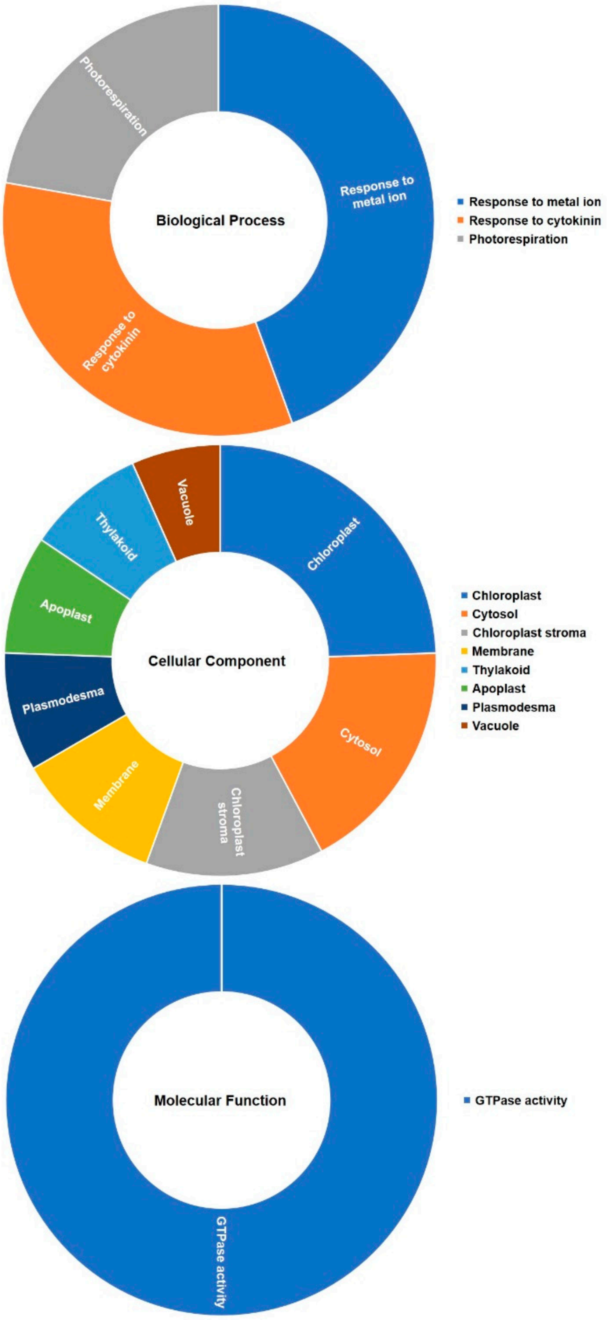

Figure S6. Significantly enriched GO terms for the DAPs between the treatment groups (CK Vs Cu50 μM) in *B. napus* seedling leaves under CA-mediated Cu stress.

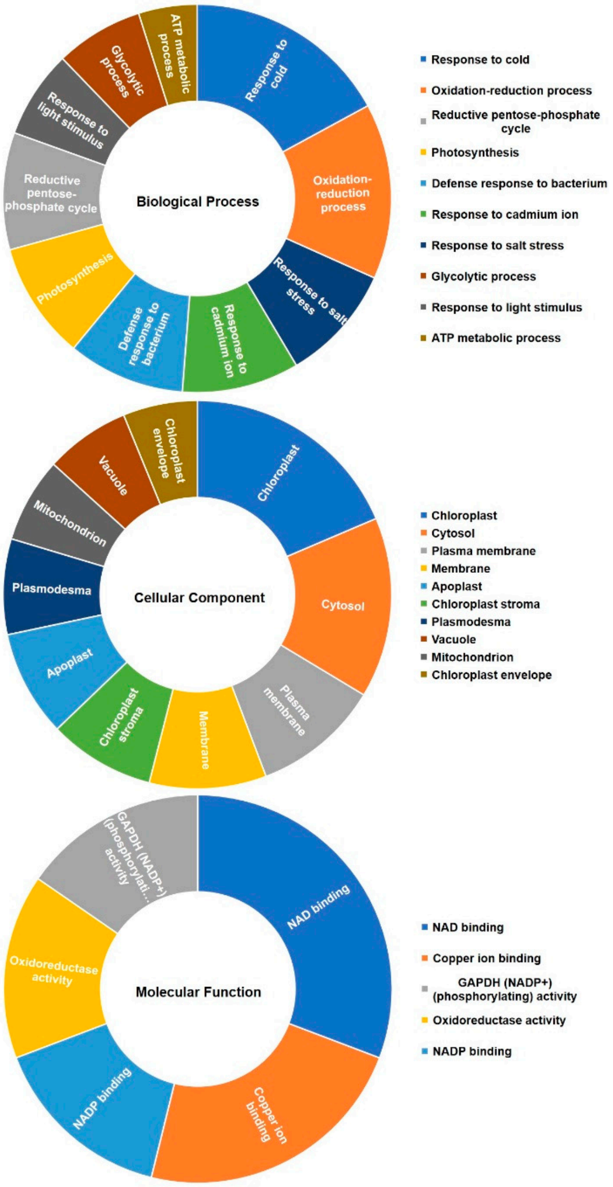

Figure S7. Significantly enriched GO terms for the DAPs between the treatment groups (CK Vs CA+Cu50 μM) in *B. napus* seedling leaves under CA-mediated Cu stress.
