# Supplementary material for: Proteome Changes Reveal the Protective Roles of Exogenous Citric Acid in Alleviating Cu Toxicity in Brassica napus L
Source: Int J Mol Sci. 2021 May 30;22(11):5879. doi: 10.3390/ijms22115879 (PMC8198124; doi:10.3390/ijms22115879)
Supplement: Supplementary file 1 [file ijms-22-05879-s001.zip › ijms-1235003-SI/Supplementary materials/Table S1.pdf]

Table S1. Primers used for qRT-PCR analysis

Protein Accession	Description	Primer	Sequence
Q8L7C9	Glutathione S-transferase U20	F	TACTTGGGTCACTGGCCATC
		R	TGGACGAATCCAGCCTATTC
Q05431	L-ascorbate peroxidase 1	F	ATGAGGTTTGACGGTGAGC
		R	CAGCATGGGAGATGGTAGG
A1E9T2	Ribulose bisphosphate carboxylase large chain	F	ACCGTTACAAAGGACGATGC
		R	CAGGGCTTTGAACCCAAATA
P27141	Carbonic anhydrase	F	AAGAGATGGGTAGCGAAGCA
		R	CGTACAAAGCAGGGTTGGTT
O49485	D-3-phosphoglycerate dehydrogenase 1	F	CTCTGGATGCGGGTATTGTT
		R	TTTCAATGGCAACTCCTTCC
P51823	ADP-ribosylation factor 2	F	ATGACAGCACTATGAC
		R	CGAGGTGATGTGAATA
