# Supplementary material for: Proteome Changes Reveal the Protective Roles of Exogenous Citric Acid in Alleviating Cu Toxicity in Brassica napus L
Source: Int J Mol Sci. 2021 May 30;22(11):5879. doi: 10.3390/ijms22115879 (PMC8198124; doi:10.3390/ijms22115879)
Supplement: Supplementary file 1 [file ijms-22-05879-s001.zip › ijms-1235003-SI/Supplementary materials/Table S5.pdf]

Table S5. KEGG pathways of the total identified proteins in *B. napus* seedlings in response to CA-mediated Cu stress using DAVID Bioinformatics

Pathway ID	Pathway Name	Number of proteins	Frequency	*p*-value	Fold Enrichment
ath01100	Metabolic pathways	260	15.84	1.21E-07	1.274708087
ath01110	Biosynthesis of secondary metabolites	147	8.96	1.91E-04	1.2964119
ath03010	Ribosome	123	7.50	1.09E-33	3.04507746
ath01130	Biosynthesis of antibiotics	106	6.46	6.26E-16	2.169907411
ath01200	Carbon metabolism	88	5.36	2.43E-23	3.018430906
ath01230	Biosynthesis of amino acids	68	4.14	2.11E-12	2.405886051
ath00630	Glyoxylate and dicarboxylate metabolism	38	2.32	4.50E-17	4.614787792
ath00190	Oxidative phosphorylation	34	2.07	3.33E-04	1.886095855
ath00710	Carbon fixation in photosynthetic organisms	33	2.01	1.07E-13	4.297983138
ath00010	Glycolysis/Gluconeogenesis	33	2.01	2.89E-07	2.624432181
ath00195	Photosynthesis	29	1.77	3.90E-09	3.384598291
ath00020	Citrate cycle (TCA cycle)	27	1.65	5.76E-10	3.851439435
ath00620	Pyruvate metabolism	27	1.65	5.85E-07	2.888579576
ath03050	Proteasome	24	1.46	1.46E-08	3.718631179
ath04145	Phagosome	22	1.34	2.82E-04	2.325967345
ath00260	Glycine, serine and threonine metabolism	21	1.28	6.87E-05	2.621118504
ath00030	Pentose phosphate pathway	18	1.10	4.24E-05	2.995564005
ath00480	Glutathione metabolism	18	1.10	0.025387323	1.739359745

ath00270	Cysteine and methionine metabolism	18	1.10	0.04354577	1.633944003
ath01210	2-Oxocarboxylic acid metabolism	17	1.04	0.006184085	2.064510328
ath00860	Porphyrin and chlorophyll metabolism	15	0.91	4.89E-04	2.808341255
ath00196	Photosynthesis - antenna proteins	13	0.79	7.08E-07	5.310318009
ath00250	Alanine, aspartate and glutamate metabolism	13	0.79	0.005063208	2.433895754
ath00051	Fructose and mannose metabolism	12	0.73	0.07690666	1.739359745
ath00220	Arginine biosynthesis	9	0.55	0.034432647	2.310863661
ath00053	Ascorbate and aldarate metabolism	9	0.55	0.077890851	1.972688491
ath00910	Nitrogen metabolism	9	0.55	0.087352042	1.925719718
ath00670	One carbon pool by folate	7	0.43	0.018109329	3.145342205
ath00261	Monobactam biosynthesis	6	0.37	0.014132619	3.851439435
ath00300	Lysine biosynthesis	6	0.37	0.019305272	3.594676806
ath00450	Selenocompound metabolism	6	0.37	0.041682918	2.995564005
