# Supplementary material for: Proteome Changes Reveal the Protective Roles of Exogenous Citric Acid in Alleviating Cu Toxicity in Brassica napus L
Source: Int J Mol Sci. 2021 May 30;22(11):5879. doi: 10.3390/ijms22115879 (PMC8198124; doi:10.3390/ijms22115879)
Supplement: Supplementary file 1 [file ijms-22-05879-s001.zip › ijms-1235003-SI/Supplementary materials/Table S6.pdf]

Table S6. Significantly enriched GO terms for the DAPs among the treatment groups in *B. napus* under CA-mediated Cu stress

GO ID	GO Name	Number of proteins	Frequency	*p*-value	Fold Enrichment
**Control vs CA**					
**Biological Process**					
GO:0046686	Response to metal ion	17	9.71	3.77E-15	15.85415406
GO:0009651	Response to stress	10	5.71	1.57E-05	6.58984041
GO:0055114	Oxidation-reduction process	10	5.71	0.01967803	2.398107337
GO:0009735	Response to cytokinin	9	5.14	8.60E-08	15.68598078
GO:0009409	Response to cold	7	4.00	3.08E-04	7.467016492
GO:0018298	Protein-chromophore linkage	6	3.43	2.58E-07	42.52643678
GO:0015979	Photosynthesis	6	3.43	6.59E-05	13.86731634
GO:0015986	ATP synthesis coupled proton transport	5	2.86	6.15E-06	40.8908046
GO:0009772	Photosynthetic electron transport in photosystem II	4	2.29	2.30E-06	141.7547893
GO:0015991	ATP hydrolysis coupled proton transport	4	2.29	2.34E-04	32.71264368
GO:0042742	Defense response to bacterium	4	2.29	0.05332936	4.622438781
GO:0006979	Response to oxidative stress	4	2.29	0.06058321	4.38416874
GO:0009765	Photosynthesis, light harvesting	3	1.71	7.12E-04	73.60344828
GO:0009768	Photosynthesis, light harvesting in photosystem I	3	1.71	0.0020711	43.49294671
GO:0010043	Response to zinc ion	3	1.71	0.01162306	18.05367599
GO:0007264	Small gtpase mediated signal transduction	3	1.71	0.04673136	8.543257389
GO:0006734	NADH metabolic process	2	1.14	0.01834812	106.316092
GO:0006107	Oxaloacetate metabolic process	2	1.14	0.01834812	106.316092
GO:0009645	Response to low light intensity stimulus	2	1.14	0.02137381	91.12807882
GO:0006094	Gluconeogenesis	2	1.14	0.04229863	45.56403941
GO:0019344	Cysteine biosynthetic process	2	1.14	0.05113127	37.52332657
GO:0006108	Malate metabolic process	2	1.14	0.05697518	33.57350272
GO:0019253	Reductive pentose-phosphate cycle	2	1.14	0.05988386	31.89482759

GO:0006913	Nucleocytoplasmic transport	2	1.14	0.0685571 2	27.73463268
GO:0019252	Starch biosynthetic process	2	1.14	0.0941079 3	19.93426724
**Cellular Component**					
GO:0009507	Chloroplast	39	22.29	6.56E-18	4.311956737
GO:0005829	Cytosol	28	16.00	1.34E-13	5.168544604
GO:0009570	Chloroplast stroma	24	13.71	8.40E-22	15.66506606
GO:0009535	Chloroplast thylakoid membrane	19	10.86	9.61E-19	19.89726398
GO:0005737	Cytoplasm	19	10.86	0.0084971 3	1.837573506
GO:0009941	Chloroplast envelope	18	10.29	3.23E-15	14.12885102
GO:0005886	Plasma membrane	18	10.29	0.0032812 8	2.072384144
GO:0005739	Mitochondrion	17	9.71	0.0035729 6	2.127347552
GO:0048046	Apoplast	16	9.14	2.78E-13	13.80470734
GO:0009506	Plasmodesma	16	9.14	5.91E-09	6.765402206
GO:0005794	Golgi apparatus	15	8.57	5.38E-07	5.214767606
GO:0016020	Membrane	13	7.43	6.98E-04	3.083396999
GO:0005618	Cell wall	11	6.29	8.61E-07	7.973509743
GO:0009579	Thylakoid	10	5.71	1.05E-09	20.69030772
GO:0005773	Vacuole	8	4.57	5.89E-04	5.42092641
GO:0009523	Photosystem II	7	4.00	4.17E-10	74.58855932
GO:0005774	Vacuolar membrane	7	4.00	0.0014436 8	5.566310397
GO:0022626	Cytosolic ribosome	6	3.43	1.08E-04	12.53589232
GO:0009534	Chloroplast thylakoid	6	3.43	1.13E-04	12.41418463
GO:0005730	Nucleolus	6	3.43	0.0030492 6	5.975051481
GO:0009536	Plastid	5	2.86	0.0052607	7.033338927
GO:0005840	Ribosome	5	2.86	0.0095479 3	5.91972693
GO:0005622	Intracellular	5	2.86	0.0584051 4	3.36135914
GO:0045261	Proton-transporting ATP synthase complex, catalytic core F(1)	3	1.71	2.32E-04	127.8661017
GO:0030076	Light-harvesting complex	3	1.71	9.67E-04	63.93305085
GO:0009522	Photosystem I	3	1.71	0.0027971 2	37.60767697
GO:0010287	Plastoglobule	3	1.71	0.0130248 6	17.04881356
GO:0022627	Cytosolic small ribosomal subunit	3	1.71	0.0294830 5	11.0229398

GO:0000275	Mitochondrial proton-transporting ATP synthase complex, catalytic core F(1)	2	1.14	0.01376044	142.0734463
GO:0000311	Plastid large ribosomal subunit	2	1.14	0.01376044	142.0734463
GO:0005753	Mitochondrial proton-transporting ATP synthase complex	2	1.14	0.03405288	56.82937853
GO:0009654	Photosystem II oxygen evolving complex	2	1.14	0.05393485	35.51836158
GO:0010319	Stromule	2	1.14	0.0798197	23.67890772
GO:0030687	Preribosome, large subunit precursor	2	1.14	0.08618138	21.85745328
**Molecular Function**					
GO:0046872	Metal ion binding	14	8.00	0.00138127	2.684783756
GO:0003735	Structural constituent of ribosome	7	4.00	0.00396527	4.538697591
GO:0016168	Chlorophyll binding	6	3.43	1.12E-07	49.94319743
GO:0046933	Proton-transporting ATP synthase activity	5	2.86	2.41E-06	51.33050847
GO:0005507	Copper ion binding	5	2.86	0.00731371	6.389689852
GO:0005525	GTP binding	5	2.86	0.01404906	5.273682378
GO:0020037	Heme binding	5	2.86	0.03464242	3.989417757
GO:0016491	Oxidoreductase activity	5	2.86	0.03605934	3.938402185
GO:0003924	GTPase activity	4	2.29	0.00882204	9.262648146
GO:0005506	Iron ion binding	4	2.29	0.09209048	3.666464891
GO:0045156	Electron transporter, transferring electrons within the cyclic electron transport pathway of photosynthesis activity	3	1.71	3.55E-04	102.6610169
GO:0031409	Pigment binding	3	1.71	0.00202232	43.99757869
GO:0046961	Proton-transporting ATPase activity, rotational mechanism	3	1.71	0.00411263	30.79830508
GO:0016615	Malate dehydrogenase activity	2	1.14	0.02525655	76.99576271
GO:0030060	L-malate dehydrogenase activity	2	1.14	0.0283692	68.44067797
GO:0070180	Large ribosomal subunit rRNA binding	2	1.14	0.06496762	29.33171913
**CK vs Cu25**					

**Biological Process**					
GO:0046686	Response to metal ion	14	12.17	1.01E-12	16.46236969
GO:0009735	Response to cytokinin	9	7.83	1.24E-08	19.77797577
GO:0009651	Response to stress	8	6.96	1.54E-04	6.64714337
GO:0006412	Translation	7	6.09	0.05287052	2.511208936
GO:0015986	ATP synthesis coupled proton transport	5	4.35	2.36E-06	51.55797101
GO:0018298	Protein-chromophore linkage	5	4.35	4.23E-06	44.68357488
GO:0009409	Response to cold	5	4.35	0.00592044	6.724952741
GO:0009772	Photosynthetic electron transport in photosystem II	4	3.48	1.12E-06	178.7342995
GO:0015991	ATP hydrolysis coupled proton transport	4	3.48	1.16E-04	41.24637681
GO:0015979	Photosynthesis	4	3.48	0.00458461	11.65658475
GO:0042742	Defense response to bacterium	4	3.48	0.02941774	5.828292376
GO:0006979	Response to oxidative stress	4	3.48	0.03364726	5.527864934
GO:0007264	Small GTPase mediated signal transduction	3	2.61	0.03036981	10.77193323
GO:0006107	Oxaloacetate metabolic process	2	1.74	0.01450886	134.0507246
GO:0006734	NADH metabolic process	2	1.74	0.01450886	134.0507246
GO:0009645	Response to low light intensity stimulus	2	1.74	0.01690691	114.9006211
GO:0009765	Photosynthesis, light harvesting	2	1.74	0.03117583	61.86956522
GO:0006094	Gluconeogenesis	2	1.74	0.03353422	57.45031056
GO:0019344	Cysteine biosynthetic process	2	1.74	0.04057574	47.31202046
GO:0006108	Malate metabolic process	2	1.74	0.04524219	42.33180778
GO:0019253	Reductive pentose-phosphate cycle	2	1.74	0.04756709	40.21521739
GO:0009768	Photosynthesis, light harvesting in photosystem I	2	1.74	0.05220029	36.55928854
GO:0006913	Nucleocytoplasmic transport	2	1.74	0.05450862	34.96975425
GO:0019252	Starch biosynthetic process	2	1.74	0.07503773	25.13451087
GO:0009567	Double fertilization forming a zygote and endosperm	2	1.74	0.08178342	22.98012422

GO:0006606	Protein import into nucleus	2	1.74	0.09733684	19.15010352
**Cellular Component**					
GO:0009507	Chloroplast	28	24.35	1.93E-11	3.886171593
GO:0005829	Cytosol	20	17.39	6.70E-09	4.634409296
GO:0009570	Chloroplast stroma	19	16.52	3.15E-17	15.56785377
GO:0009941	Chloroplast envelope	16	13.91	2.36E-14	15.76552643
GO:0005737	Cytoplasm	16	13.91	0.00960456	1.942518913
GO:0009535	Chloroplast thylakoid membrane	15	13.04	1.01E-14	19.71901302
GO:0005794	Golgi apparatus	13	11.30	1.42E-06	5.673371282
GO:0048046	Apoplast	12	10.43	1.08E-09	12.9969851
GO:0005739	Mitochondrion	12	10.43	0.04006438	1.885058907
GO:0009506	Plasmodesma	9	7.83	4.36E-04	4.777165653
GO:0009579	Thylakoid	8	6.96	9.13E-08	20.77835158
GO:0016020	Membrane	8	6.96	0.04345573	2.381936798
GO:0005618	Cell wall	7	6.09	6.74E-04	6.369554205
GO:0009523	Photosystem II	6	5.22	1.03E-08	80.25638298
GO:0005840	Ribosome	5	4.35	0.00419731	7.431146572
GO:0022626	Cytosolic ribosome	4	3.48	0.00617928	10.49103045
GO:0009534	Chloroplast thylakoid	4	3.48	0.0063476	10.38917579
GO:0009536	Plastid	4	3.48	0.01795153	7.063268029
GO:0005774	Vacuolar membrane	4	3.48	0.07457782	3.992854875
GO:0045261	Proton-transporting ATP synthase complex, catalytic core F(1)	3	2.61	1.46E-04	160.512766
GO:0005747	Mitochondrial respiratory chain complex I	3	2.61	0.00524162	27.20555355
GO:0005623	Cell	3	2.61	0.04820493	8.360039894
GO:0000311	Plastid large ribosomal subunit	2	1.74	0.01092648	178.3475177
GO:0000275	Mitochondrial proton-transporting ATP synthase complex, catalytic core F(1)	2	1.74	0.01092648	178.3475177
GO:0005753	Mitochondrial proton-transporting atp synthase complex	2	1.74	0.02709753	71.33900709
GO:0030076	Light-harvesting complex	2	1.74	0.03596939	53.50425532
GO:0009522	Photosystem i	2	1.74	0.06039168	31.47309136

GO:0010319	stromule	2	1.74	0.0638308	29.72458629
GO:0030687	Preribosome, large subunit precursor	2	1.74	0.06896639	27.43807965
GO:0015935	Small ribosomal subunit	2	1.74	0.08923459	20.98206091
**Molecular Function**					
GO:0046872	Metal ion binding	9	7.826086957	0.04154309	2.213695923
GO:0003735	Structural constituent of ribosome	7	6.086956522	0.00106074	5.821372998
GO:0046933	Proton-transporting ATP synthase activity	5	4.347826087	8.58E-07	65.83695652
GO:0016168	Chlorophyll binding	5	4.347826087	2.04E-06	53.3813161
GO:0005507	Copper ion binding	4	3.47826087	0.02171786	6.556377413
GO:0005506	Iron ion binding	4	3.47826087	0.05027946	4.702639752
GO:0016491	Oxidoreductase activity	4	3.47826087	0.07231973	4.041143111
GO:0045156	Electron transporter, transferring electrons within the cyclic electron transport pathway of photosynthesis activity	3	2.608695652	2.14E-04	131.673913
GO:0046961	Proton-transporting ATPase activity, rotational mechanism	3	2.608695652	0.00249609	39.50217391
GO:0030170	Pyridoxal phosphate binding	3	2.608695652	0.03189686	10.48730281
GO:0019843	rRNA binding	3	2.608695652	0.04885652	8.287169352
GO:0016615	Malate dehydrogenase activity	2	1.739130435	0.01964467	98.75543478
GO:0030060	L-malate dehydrogenase activity	2	1.739130435	0.02207356	87.7826087
GO:0070180	Large ribosomal subunit rRNA binding	2	1.739130435	0.05076526	37.62111801
GO:0031409	Pigment binding	2	1.739130435	0.05076526	37.62111801
**CK vs CA+Cu25**					
**Biological Process**					
GO:0046686	Response to metal ion	13	12.38	8.84E-12	16.35298518
GO:0055114	Oxidation-reduction process	7	6.67	0.07807391	2.264259486
GO:0015979	Photosynthesis	4	3.81	0.0037698	12.46983485
GO:0009735	Response to cytokinin	4	3.81	0.00823711	9.403482018

GO:0042742	Defense response to bacterium	4	3.81	0.0245832<br>4	6.234917425
GO:0006457	Protein folding	4	3.81	0.0274284<br>2	5.975129199
GO:0009793	Embryo development ending in seed dormancy	4	3.81	0.0856659<br>9	3.749100674
GO:0019253	Reductive pentose-phosphate cycle	3	2.86	9.32E-04	64.53139535
GO:0009853	Photorespiration	3	2.86	0.0057533<br>5	25.81255814
GO:0006099	Tricarboxylic acid cycle	3	2.86	0.0064441<br>8	24.35146994
GO:0009416	Response to light stimulus	3	2.86	0.0678818<br>3	6.865042058
GO:0046034	ATP metabolic process	2	1.90	0.0157874<br>5	122.9169435
GO:0015977	Carbon fixation	2	1.90	0.0180228<br>5	107.5523256
GO:0046688	Response to copper ion	2	1.90	0.0466358<br>2	40.97231451
GO:0007017	Microtubule-based process	2	1.90	0.0574227	33.09302326
GO:0015991	ATP hydrolysis coupled proton transport	2	1.90	0.0849140<br>4	22.0620155
**Cellular Component**					
GO:0009507	Chloroplast	31	29.52	4.14E-15	4.493771437
GO:0005829	Cytosol	26	24.76	2.64E-15	6.292497955
GO:0009570	Chloroplast stroma	19	18.10	1.21E-17	16.25975838
GO:0048046	Apoplast	17	16.19	9.41E-17	19.23072425
GO:0005737	Cytoplasm	14	13.33	0.0346104<br>3	1.775246451
GO:0016020	Membrane	13	12.38	4.19E-05	4.042676065
GO:0005739	Mitochondrion	13	12.38	0.0119660<br>6	2.132909245
GO:0005886	Plasma membrane	13	12.38	0.0222074<br>9	1.96236869
GO:0005794	Golgi apparatus	12	11.43	6.14E-06	5.4697118
GO:0009506	Plasmodesma	11	10.48	7.34E-06	6.098258377
GO:0009535	Chloroplast thylakoid membrane	10	9.52	3.00E-08	13.73027573
GO:0009941	Chloroplast envelope	10	9.52	3.46E-07	10.29138531
GO:0009579	Thylakoid	9	8.57	2.44E-09	24.41456311
GO:0005773	Vacuole	8	7.62	1.02E-04	7.107436849
GO:0005774	Vacuolar membrane	7	6.67	3.24E-04	7.29805141
GO:0005618	Cell wall	7	6.67	5.28E-04	6.652645503
GO:0009534	Chloroplast thylakoid	6	5.71	2.94E-05	16.2763754
GO:0022626	Cytosolic ribosome	5	4.76	4.43E-04	13.69662309
GO:0009536	Plastid	5	4.76	0.0019196	9.221488816

GO:0009543	Chloroplast thylakoid lumen	4	3.81	3.74E-04	27.94111111
GO:0031977	Thylakoid lumen	3	2.86	0.00361268	32.87189542
GO:0005759	Mitochondrial matrix	3	2.86	0.01537279	15.52283951
GO:0033180	Proton-transporting V-type ATPase, V1 domain	2	1.90	0.01045349	186.2740741
GO:0030095	Chloroplast photosystem II	2	1.90	0.02934156	65.74379085
**Molecular Function**					
GO:0005524	ATP binding	12	11.42857143	0.01647907	2.137848543
GO:0005507	Copper ion binding	6	5.714285714	2.24E-04	10.52069864
GO:0005525	GTP binding	6	5.714285714	5.41E-04	8.683179356
GO:0003924	GTPase activity	5	4.761904762	2.47E-04	15.88651862
GO:0046933	Proton-transporting ATP synthase activity	3	2.857142857	0.00217752	42.25813953
GO:0051287	NAD binding	3	2.857142857	0.01235561	17.36635871
GO:0016984	Ribulose-bisphosphate carboxylase activity	2	1.904761905	0.01150484	169.0325581
GO:0002020	Protease binding	2	1.904761905	0.03188879	60.36877076
GO:0008266	Poly(U) RNA binding	2	1.904761905	0.0430353	44.48225214
GO:0046961	Proton-transporting ATPase activity	2	1.904761905	0.06711845	28.17209302
GO:0005200	Structural constituent of cytoskeleton	2	1.904761905	0.09481821	19.65494862
**CK vs Cu50**					
**Biological Process**					
GO:0046686	Response to metal ion	4	5.26	0.00241612	13.52266082
GO:0009735	Response to cytokinin	3	3.95	0.00938991	18.95389344
GO:0009853	Photorespiration	2	2.63	0.03979936	46.2475
**Cellular Component**					
GO:0009507	Chloroplast	11	14.47	1.01E-05	4.484711414
GO:0005829	Cytosol	8	10.53	1.81E-04	5.445430922
GO:0009570	Chloroplast stroma	6	7.89	2.81E-05	14.44123277
GO:0016020	Membrane	5	6.58	0.01875624	4.37308709

GO:0009579	Thylakoid	4	5.26	2.29E-04	30.51820388
GO:0048046	Apoplast	4	5.26	0.00287519	12.72621457
GO:0009506	Plasmodesma	4	5.26	0.02036638	6.236855159
GO:0005773	Vacuole	3	3.95	0.0528944	7.496124801
**Molecular Function**					
GO:0003924	GTPase activity	2	2.63	0.09776906	18.21654135
**CK vs Cu50**					
**Biological Process**					
GO:0009409	Response to cold	7	6.80	3.77E-06	15.4673913
GO:0055114	Oxidation-reduction process	6	5.83	0.04088999	2.980504834
GO:0019253	Reductive pentose-phosphate cycle	4	3.88	3.11E-06	132.1357143
GO:0015979	Photosynthesis	4	3.88	0.00104206	19.15010352
GO:0042742	Defense response to bacterium	4	3.88	0.00737362	9.57505176
GO:0046686	Response to cadmium ion	4	3.88	0.01319037	7.727234754
GO:0009651	Response to stress	4	3.88	0.03269294	5.460153483
GO:0006096	Glycolytic process	3	2.91	0.0047912	27.91599598
GO:0009416	Response to light stimulus	3	2.91	0.03052937	10.54274316
GO:0046034	ATP metabolic process	2	1.94	0.01017378	188.7653061
GO:0006006	Glucose metabolic process	2	1.94	0.03305524	57.45031056
GO:0015991	ATP hydrolysis coupled proton transport	2	1.94	0.05542691	33.88095238
GO:0009744	Response to sucrose	2	1.94	0.07323607	25.41071429
GO:0006099	Tricarboxylic acid cycle	2	1.94	0.07459253	24.93126685
**Cellular Component**					
GO:0009507	Chloroplast	21	20.39	1.46E-11	4.892412451
GO:0005829	Cytosol	17	16.50	1.18E-10	6.612308977
GO:0005886	Plasma membrane	12	11.65	9.16E-04	2.911206298
GO:0016020	Membrane	11	10.68	9.24E-06	5.497595198
GO:0048046	Apoplast	10	9.71	1.39E-09	18.18030654
GO:0009570	Chloroplast stroma	10	9.71	1.57E-08	13.75355502
GO:0009506	Plasmodesma	9	8.74	7.29E-06	8.018813776
GO:0005773	Vacuole	8	7.77	3.40E-06	11.42266636

GO:0005739	Mitochondrion	8	7.77	0.0633090 7	2.109470682
GO:0009941	Chloroplast envelope	7	6.80	1.98E-05	11.57780847
GO:0005794	Golgi apparatus	7	6.80	0.0016234 6	5.127854812
GO:0010319	Stromule	6	5.83	3.55E-10	149.6845238
GO:0009579	Thylakoid	6	5.83	2.45E-06	26.15846047
GO:0009535	Chloroplast thylakoid membrane	6	5.83	6.52E-05	13.23990874
GO:0005774	Vacuolar membrane	6	5.83	2.36E-04	10.05343817
GO:0033180	Proton-transporting V-type ATPase, V1 domain	2	1.94	0.0064254 9	299.3690476
**Molecular Function**					
GO:0051287	NAD binding	4	3.88	1.70E-04	35.55968689
GO:0005507	Copper ion binding	3	2.91	0.0494781 9	8.078393598
GO:0047100	Glyceraldehyde-3-phosphate dehydrogenase (NADP+) (phosphorylating) activity	2	1.94	0.0044512 8	432.6428571
GO:0016620	Oxidoreductase activity, acting on the aldehyde or oxo group of donors, NAD or NADP as acceptor	2	1.94	0.0147635 1	129.7928571
GO:0050661	NADP binding	2	1.94	0.0827553 3	22.37807882
