# Supplementary material for: Proteome Changes Reveal the Protective Roles of Exogenous Citric Acid in Alleviating Cu Toxicity in Brassica napus L
Source: Int J Mol Sci. 2021 May 30;22(11):5879. doi: 10.3390/ijms22115879 (PMC8198124; doi:10.3390/ijms22115879)
Supplement: Supplementary file 1 [file ijms-22-05879-s001.zip › ijms-1235003-SI/Supplementary materials/Table S7.pdf]

Table S7. List of significantly enriched KEGG pathways for the DAPs among the treatment groups in *B. napus seedlings in response to CA-mediated Cu stress using DAVID Bioinformatics.*

| Pathway ID | Pathway Name | Number of proteins | Frequency | *p*-value | Fold Enrichment |
|---|---|---|---|---|---|
| **CK vs CA** | | | | | |
| ath01100 | Metabolic pathways | 31 | 17.71 | 0.002809825 | 1.537380922 |
| ath01200 | Carbon metabolism | 10 | 5.71 | 0.001677374 | 3.469612449 |
| ath01130 | Biosynthesis of antibiotics | 10 | 5.71 | 0.042739555 | 2.070702646 |
| ath00195 | Photosynthesis | 8 | 4.57 | 1.50E-05 | 9.444555445 |
| ath00710 | Carbon fixation in photosynthetic organisms | 5 | 2.86 | 0.006202895 | 6.587235229 |
| ath00630 | Glyoxylate and dicarboxylate metabolism | 5 | 2.86 | 0.007936414 | 6.142151767 |
| ath00270 | Cysteine and methionine metabolism | 5 | 2.86 | 0.021329601 | 4.591103341 |
| ath00190 | Oxidative phosphorylation | 5 | 2.86 | 0.095981685 | 2.805674264 |
| ath00196 | Photosynthesis - antenna proteins | 3 | 1.71 | 0.022973593 | 12.39597902 |
| **CK vs Cu25** | | | | | |
| ath01100 | Metabolic pathways | 28 | 24.35 | 6.88E-04 | 1.679239777 |
| ath01130 | Biosynthesis of antibiotics | 8 | 6.96 | 0.089396938 | 2.00328442 |
| ath00195 | Photosynthesis | 7 | 6.09 | 5.05E-05 | 9.993657505 |
| ath01200 | Carbon metabolism | 7 | 6.09 | 0.026733025 | 2.937067282 |
| ath00190 | Oxidative phosphorylation | 6 | 5.22 | 0.013568002 | 4.071490095 |
| ath00710 | Carbon fixation in photosynthetic organisms | 5 | 4.35 | 0.00306524 | 7.965958881 |
| ath00630 | Glyoxylate and dicarboxylate metabolism | 4 | 3.48 | 0.027329023 | 5.942174733 |
| ath00270 | Cysteine and methionine metabolism | 4 | 3.48 | 0.0568231 | 4.441625558 |
| **CK vs CA+Cu25** | | | | | |
| ath01200 | Carbon metabolism | 13 | 12.38 | 1.74E-07 | 6.515161154 |
| ath00630 | Glyoxylate and dicarboxylate metabolism | 8 | 7.62 | 8.19E-07 | 14.1951952 |

| | | | | | |
|---|---|---|---|---|---|
| ath01100 | Metabolic pathways | 25 | 23.81 | 3.28E-04 | 1.790855913 |
| ath00710 | Carbon fixation in photosynthetic organisms | 5 | 4.76 | 0.001548155 | 9.51489533 |
| ath01130 | Biosynthesis of antibiotics | 10 | 9.52 | 0.003725546 | 2.991014933 |
| ath00260 | Glycine, serine and threonine metabolism | 4 | 3.81 | 0.015647735 | 7.294753086 |
| ath00195 | Photosynthesis | 4 | 3.81 | 0.018716314 | 6.821067821 |
| ath04145 | Phagosome | 4 | 3.81 | 0.024277485 | 6.179084967 |
| ath01230 | Biosynthesis of amino acids | 6 | 5.71 | 0.037309398 | 3.101706037 |
| ath00020 | Citrate cycle (TCA cycle) | 3 | 2.86 | 0.078557242 | 6.252645503 |
| **CK vs Cu50** | | | | | |
| ath01100 | Metabolic pathways | 8 | 10.53 | 0.084866235 | 1.719221677 |
| **CK vs CA+Cu50** | | | | | |
| ath01200 | Carbon metabolism | 8 | 7.77 | 1.23E-04 | 6.275472951 |
| ath00710 | Carbon fixation in photosynthetic organisms | 5 | 4.85 | 2.50E-04 | 14.89287965 |
| ath01100 | Metabolic pathways | 15 | 14.56 | 0.015775682 | 1.681847292 |
| ath04145 | Phagosome | 3 | 2.91 | 0.058510303 | 7.25370844 |
| ath00010 | Glycolysis/Gluconeogenesis | 3 | 2.91 | 0.095980436 | 5.456329357 |
